# The Immediate Early Gene Product EGR1 and Polycomb Group Proteins Interact in Epigenetic Programming during Chondrogenesis

**DOI:** 10.1371/journal.pone.0058083

**Published:** 2013-03-06

**Authors:** Frank Spaapen, Guus G. H. van den Akker, Marjolein M. J. Caron, Peggy Prickaerts, Celine Rofel, Vivian E. H. Dahlmans, Don A. M. Surtel, Yvette Paulis, Finja Schweizer, Tim J. M. Welting, Lars M. Eijssen, Jan Willem Voncken

**Affiliations:** 1 Department of Molecular Genetics, Maastricht University Medical Centre, Maastricht, The Netherlands; 2 Department of Orthopaedic Surgery, Maastricht University Medical Centre, Maastricht, The Netherlands; 3 Department of Bioinformatics – BiGCaT, Maastricht University Medical Centre, Maastricht, The Netherlands; North Carolina State University, United States of America

## Abstract

Initiation of and progression through chondrogenesis is driven by changes in the cellular microenvironment. At the onset of chondrogenesis, resting mesenchymal stem cells are mobilized *in vivo* and a complex, step-wise chondrogenic differentiation program is initiated. Differentiation requires coordinated transcriptomic reprogramming and increased progenitor proliferation; both processes require chromatin remodeling. The nature of early molecular responses that relay differentiation signals to chromatin is poorly understood. We here show that immediate early genes are rapidly and transiently induced in response to differentiation stimuli *in vitro*. Functional ablation of the immediate early factor EGR1 severely deregulates expression of key chondrogenic control genes at the onset of differentiation. In addition, differentiating cells accumulate DNA damage, activate a DNA damage response and undergo a cell cycle arrest and prevent differentiation associated hyper-proliferation. Failed differentiation in the absence of EGR1 affects global acetylation and terminates in overall histone hypermethylation. We report novel molecular connections between EGR1 and Polycomb Group function: Polycomb associated histone H3 lysine27 trimethylation (H3K27me3) blocks chromatin access of EGR1. In addition, EGR1 ablation results in abnormal *Ezh2* and *Bmi1* expression. Consistent with this functional interaction, we identify a number of co-regulated targets genes in a chondrogenic gene network. We here describe an important role for EGR1 in early chondrogenic epigenetic programming to accommodate early gene-environment interactions in chondrogenesis.

## Introduction

Differentiation requires orchestration of numerous parallel cellular responses and altered physiological states associated with the novel cell fate. Such changes are often induced by environmental cues (*i.a.* soluble factors, cell-cell contacts), that are transduced to the nucleus and translated into spatio-temporal reprofiling of gene expression. On their way to becoming terminally differentiated chondrocytes, chondrogenic progenitor cells undergo a well-described sequential series of events at the cell biology level: initially resting growth plate stem cells undergo a transient replicative burst. *In vivo*, this rapid progenitor expansion in the so-called proliferative zone is a distinctive feature of developing cartilage. During endochondral ossification, as cells move away from the growth plate, they simultaneously differentiate, become hypertrophic and are ultimately replaced by mineralized bone tissue [1], [2]. Chondrogenesis is controlled by numerous well-described environmental and endocrine factors [1]–[6]. Per example, signaling through the insulin receptor has been intensively studied because of its mobilizing effect on resting stem cells and stimulatory effects on cells in the proliferative zone [7]. Progression through chondrogenesis is in part driven by interaction with a constantly changing microenvironment, which is defined by soluble growth and differentiation factors, hormones, oxygen tension, cell-cell and cell-ECM contacts [3], [4], [6], [8]. Cells respond to these changes in the microenvironment by altering their transcriptome [9], [10]. Post-translational modification of histone tails serves to recruit transcriptional activators or repressors and/or nucleosome remodeling machineries, and as such constitutes an epigenetic register of expression potential [11]–[13]. Although pathways and mechanisms involved in chondrogenesis are continuously being defined, important issues surrounding the most primary steps in chondrogenic commitment and differentiation remain to be elucidated. This includes what connects environmental cues to chromatin and which signaling factors are involved in early epigenomic remodeling and, hence, in differentiation.

Polycomb Repressive Complexes (PRCs; PRC1 and PRC2) are important factors in cell fate determination: PRCs provide cells with an epigenetic memory function. An increasing number of studies links Polycomb function to important developmental processes and provide evidence for regulation of PRCs by multiple signaling pathways [14], [15]. Relevant to the study herein: many single and compound PRC1 loss-of function (LOF) mouse models display antero-posterior (AP) segmentation abnormalities due to defective *Hox* gene expression boundary maintenance within the *Hox*-clusters. The abnormal skeletogenesis in PRC1 LOF mice suggests a potential direct link to endochondral ossification (*i.e.* formation of an ossified skeleton from a cartilagenous scaffold) [16].

The first line of cellular responses to environmental and intrinsic stimuli involves rapid activation of immediate early genes (IEG). As such IEGs also represent an important gateway to genomic responses and physiological adaptation. Although, their connection to skeletogenesis is relatively poorly understood, IEGs encoding c-FOS and c-JUN are implicated in different aspects of bone physiology [17], [18]. The gene products of Early Growth Response (*EGR*) gene family, *EGR1 (KROX24/NGFI-A/TIS8/zif268), EGR2 (KROX20, CMT4E), EGR3 and EGR4 (NGFI-C)* have been implicated in several neuro-muscular and musculo-skeletal processes [19]–[23]. Molecular genetic mouse models support a potential pleiotropic regulatory function for EGR1 in endochondral ossification, based on abnormal fracture callus formation and mineralization [24], [25], but fail to pinpoint a role for EGR1 in chondrogenesis, most likely due to redundant action of EGR family proteins. Although these observations support a potential role for EGR1 in cartilage physiology, a role for EGR1 in chondrogenesis remained to be elucidated. Our and other laboratories have established a connection between IEG responses and PRC function [26], [27]. We therefore also probed a possible link between IEG and PRC function in chondrogenesis.

Using RNA interference-mediated depletion of EGR1 in a chondrogenic cell model *in vitro*, we here report that IEG family members (*i.a. Fos, Jun, Egr*) are rapidly induced in response to insulin signaling. We show that *Egr1* mRNA induction in chondrogenesis is transient and precedes transcriptional upregulation of *Sox9*, an established key-regulator of chondrogenesis. Acute loss of EGR1 prevents *Sox9* induction, in support of a role for EGR1 in transcriptional regulation of *Sox9*. In addition, loss of EGR1 leads to replication arrest in culture, which correlates with increased expression of genes associated with DNA damage response and cell cycle arrest. We show that early EGR1 depletion has long-lasting effects on epigenomic reprogramming and describe a novel functional link between EGR1 and Polycomb Repressive Complexes, providing at least in part an explanation for the observed defective differentiation in the EGR1-ablation model.

## Results

### Egr1 is rapidly induced in chondrogenesis

**Figure 1:**
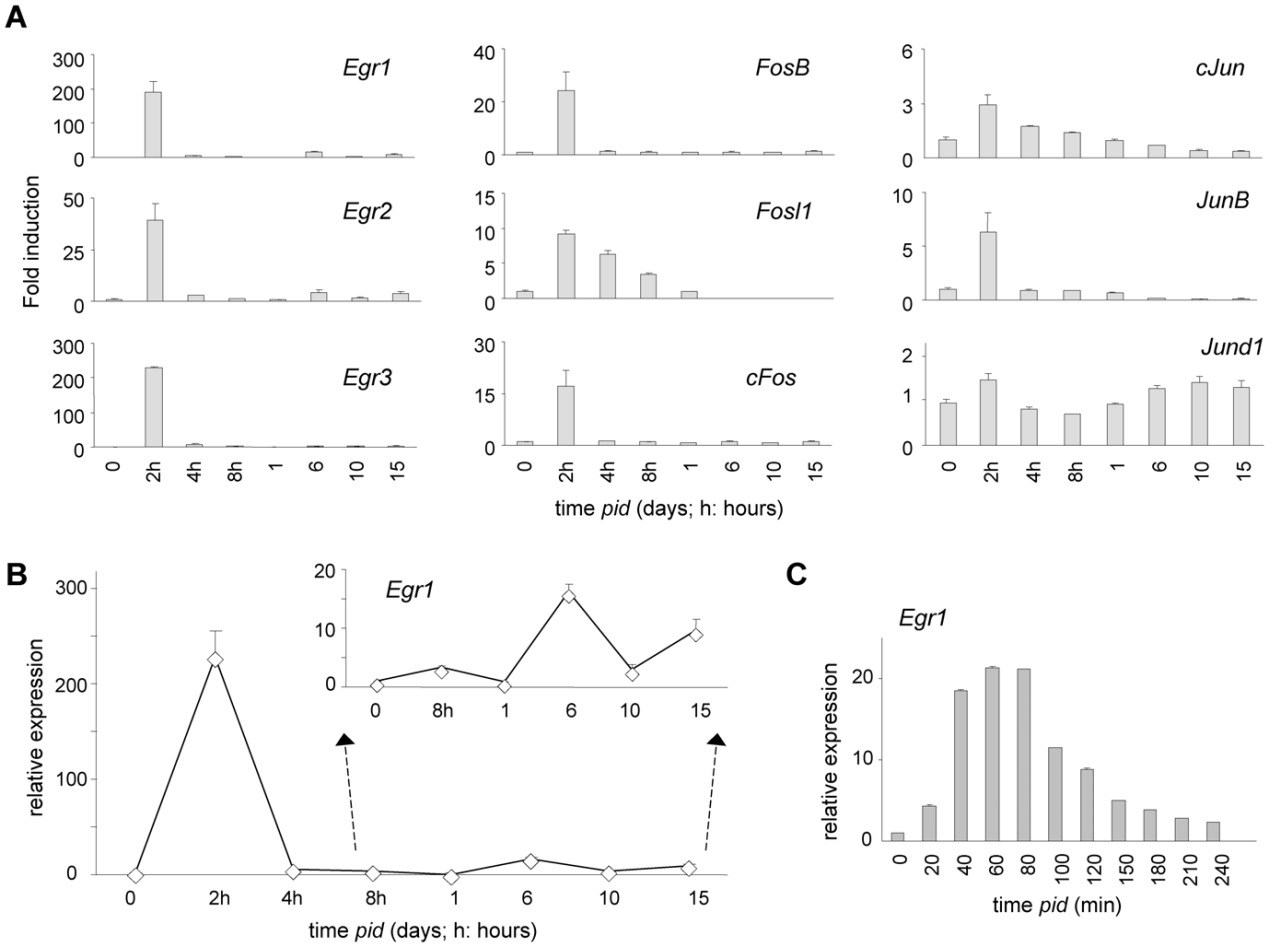
Induction of Immediate early gene expression during chondrogenesis in ATDC5 cultures. (A) Induction of *Egr*, *Fos* and *Jun* family members; expression *IEG* mRNA expression in chondrogenic differentiation presented as fold induction compared to t = 0; data are based on array analysis of three independent replicate RNA samples (normalization was done against *cyclophyllin A*); (B) Biphasic *Egr1* expression profile during chondrogenesis, inset magnifies t = 8 hours until t = 10 days *pid* (relative expression level presented in arbitrary units; for normalization see detailed description in *Methods* section). C) qPCR analysis of *Egr1 expression* (mRNA) up to 4 hours *pid* at indicated time intervals; standard error is based on three independent, parallel experimental samples; expression was normalized to *cyclophilin*.

To identify immediate early growth response gene (IEG) activation in response to chondrogenic stimuli, murine ATDC5 cells were stimulated to differentiate using medium supplemented with Insulin, Transferrin and selenite (ITS; see *Methods*). ATDC5 cells were originally identified as stem cells of mesenchymal origin that harbor chondrogenic potential [28]. ATDC5 differentiation recapitulates relevant chondrogenic features *in vitro*, including timed transcriptomic re-profiling, increased proliferation and formation of chondrogenic nodules [28]–[33]; hence, ATDC5 cells represent an excellent model to study the effect of chondrogenic differentiation stimuli on gene expression at the epigenetic level. An expression-array experiment was designed to assay immediate early, early and late changes in gene expression profiles, as a function of time (0, 2, 4, 8, 16, 24 and 72 hrs). Expression array analysis revealed rapid upregulation of a number of IEGs: expression of genes belonging to the *Fos*, *Jun* and *Egr* subfamilies (*Egr1, Egr2, Egr3, FosB, Fosl1, cFos, cJun, JunB, JunD*) was significantly enhanced within 2 hours *post-induction of differentiation* (*pid*) (Figure 1A). The IEG *c-Myc* appeared already expressed in undifferentiated ATDC5; consequently *c-Myc* mRNA induction was relatively moderate (<2x; data not show). Expression of most IEGs rapidly declined over the next 2 hours. To investigate a role for IEGs in chondrogenesis we focused on the *Egr* family; *Egr1* and *Egr3* were both massively induced upon adding differentiation medium: *Egr1* mRNA expression reached its highest level within two hours of stimulation and returned to baseline levels at 4 hours *pid*. *Egr1* expression increased again later during chondrogenesis around 6 days (Figure 1B). An independent quantitative (qPCR) expression analysis using a time resolution of 20 minute intervals essentially confirmed the microarray findings: *Egr1* mRNA increased within 20 minutes into differentiation, peaked at 1–2 hours *pid* and dropped to pre-induction levels within 2–3 hours (Figure 1C). The early transient IEG-induction profile suggested a role for EGR1 in activating downstream differentiation programs.

### EGR1 controls chondrogenesis through SOX9 and RUNX2

**Figure 2:**
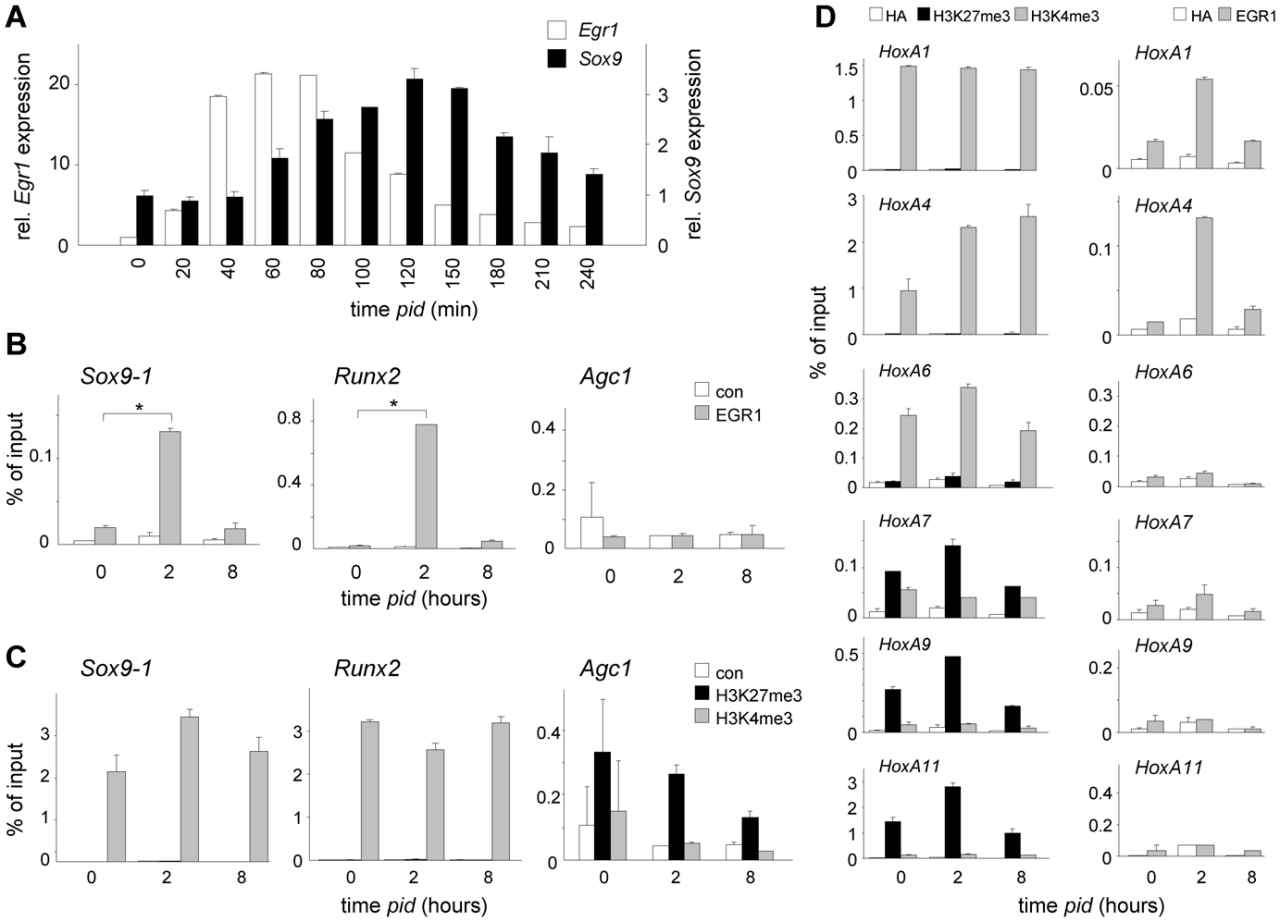
EGR1 targets chondrogenic key regulators. (A) Out-of-phase expression of *Egr1* and *Sox9* during early chondrogenesis; qPCR analysis of *Egr1 and Sox9* mRNA (until 4 hours *pid*) at indicated time intervals; standard error is based on three independent, parallel experiments; expression was normalized to *cyclophilin A*; expression profile *Egr1 cf.* Figure 1C. (B) Analysis of EGR1 occupation at *Sox9*, *Runx2* and *Agc1* promoters indicated promoters at 0, 2 and 8 hours *pid*: *: P values (EGR1/chromatin enrichment at t = 2 vs t = 0): 0.021, 0.038 and 0.88, respectively. (C) H3K27me3 and H3K4me3 enrichment on indicated promoters at 0, 2 and 8 hours *pid*. Control (con) ChIP experiments were carried out with a non-relevant haemagglutinin (HA) anti-serum. (D) Correlative H3K4me3 marking and (left) and EGR1/chromatin binding (right) within the *HoxA* cluster. ChIP analysis for enrichment of H3K27me3, H3K4me3 and Egr1 on indicated *HoxA* gene promoters; control (con) ChIP experiments were carried out with a non-relevant haemagglutinin (HA) anti-serum.

To study a potential regulatory function of EGR1 in chondrogenesis, we analysed the effect of EGR1 knock-down (KD) on *Sox9* and *Runx2* expression, both known key regulators of chondrogenesis [34]–[37]. At the mRNA level, *Sox9* induction followed that of *Egr1* approximately 1 hour out-of-phase in response to differentiation medium (Figure 2A), and increased again from 6 days *pid* onwards (Figure S1A; *cf.* Figure 1B) [38]. The out-of-phase *Sox9*-induction kinetics were consistent with transcriptional regulation by EGR1. *In silico Sox9* promoter analysis revealed several putative binding sites for EGR family members a number of which correspond to potential EGR1-consensus binding sites (Figure S1B). To obtain experimental evidence for direct EGR1 binding, we performed chromatin immunoprecipitation (ChIP) with anti-EGR1 antibodies on ATDC5 cell extracts differentiated for 0, 2 and 8 hours. Two independent primer sets were designed to detect co-precipitated *Sox9* promoter sequences using real-time PCR (Figure S1B). In good agreement with the expression kinetics of *Egr1*, both *Sox9* primer sets showed substantial enrichment of EGR1-protein within the vicinity of the EGR1 binding sites at 2 hours *pid* (10–15 fold; Figure 2B; Figure S1C,D). Likewise, the *Runx2* promoter was enriched 67-fold for EGR1 at 2 hours *pid* (Figure 2B). Both promoters carried histone H3K4me3, reflecting an epigenetic status permissive for transcriptional activation (Figure 2C). Consistent with a transient rise and fall in EGR1 protein levels early in differentiation (see below), EGR1 occupation at these promoters was restored at 8 hours *pid* to levels found in undifferentiated cells (Figure 2B,C; Figure S1C). Conversely, the Aggrecan (*Agc1*) promoter, which carries potential EGR1 binding sites (*cf.* Figure S1B), appeared not enriched for EGR1 at 2 hours *pid*, and, in contrast to the *Sox9* and *Runx2* promoters, the *Agc1* promoter was marked for repression by H3K27me3 (Figure 2B,C). Both findings were consistent with a significantly later transcriptional activation of *Agc1* during chondrogenesis, as opposed to *Sox9* and *Runx2*, at approximately 10 days (Figure S1A, Figure S2A,B) and suggested that local H3K27me3 limited access of EGR1 to chromatin. Similarly, despite the presence of consensus EGR1-binding sequences in its promoter (*cf.* Figure S1B), EGR1-enrichment in the *Sox6*-promoter was only marginally changed and *shEgr1* appeared to have little effect on *Sox6*-mRNA induction (Figure S3A,C); this was consistent with the absence of any effect of EGR1-depletion on *Sox6*-expression between 0 and 24 hrs *pid* (Figure S3B). Analogous to the *Agc1*-promoter,the *Sox6*-promotor appeared enriched for H3K27me3 in ATDC5 cells (Figure S3D). These findings suggested that the *Sox6* and *Agc1*-loci do not represent early EGR1-targets for transcriptional activation in chondrogenesis. Interestingly, local H3K27me3 was reduced overtime and both *Sox6* and *Agc1* are expressed later in chondrogenesis (Figure S3A,B); suggesting a potential regulatory role for PRC1-mediated repression of chondrogenic genes early in differentiation. In contrast, *Sox4* was rapidly occupied by EGR1, but not expressed until past 24 hours into differentiation, indicating that EGR1 binding *per se* is not sufficient for transcriptional activation (Figure S4A–C; *cf.* Figure S1B).

To independently establish the relationship between EGR1/chromatin binding and pre-existing epigenetic marking, we exploited the *Hox* gene clusters. Expression of *Hox* genes is in part controlled by PRCs in the context of AP-development: PRC-mediated repression (*i.e.* H3K27me3-marking) defines expression boundaries within the respective *Hox* clusters (*HoxA*, *B*, *C*, *D*) [39]–[42]. Relevantly, *Hox* clusters, including *HoxA*, carry predicted EGR1 binding sites (not shown) and can thus be used to study EGR1/chromatin binding. Comparative array analysis showed that expression (mRNA) of *Hox* genes within the *A*, *B*, *C* and *D* clusters remained largely unaltered throughout chondrogenesis and that their expression was not affected by loss of EGR1 (Figure S5). The direct implication of this observation was that EGR1/chromatin binding throughout the cluster could be assessed independent of additional transcriptional regulation. We analyzed *HoxA* cluster genes *A1*, *A4*, *A6*, *A7*, *A9* and *A11* for EGR1 occupation and for pre-existing H3K27me3 and H3K4me3-marks. The *HoxA* gene cluster showed the expected bimodal distribution of H3K4me3 and H3K27me3 marks: the proximal *HoxA1* and *HoxA4* promoters were up to 400-fold enriched for H3K4me3, whereas none of these promoters were enriched for H3K27me3. In contrast, the distal *HoxA9* and *HoxA11* promoters lacked H3K4me3, but instead showed enrichment for the repressive H3K27me3 mark (Figure 2D). Of note, HoxA6 and HoxA7 promoters showed moderate enrichment for both opposing trimethyl marks, thus positioning the PRC-dependent expression boundary within the *HoxA* cluster approximately at *HoxA6/A7* in non-differentiated ATDC5 cells. Strikingly, EGR1 was only enriched at accessible H3K4me3-marked *HoxA* promoters; in clear contrast, none of the H3K27me3-marked *HoxA* promoters were EGR1-enriched at 2 hours into differentiation (Figure 2D).

**Figure 3:**
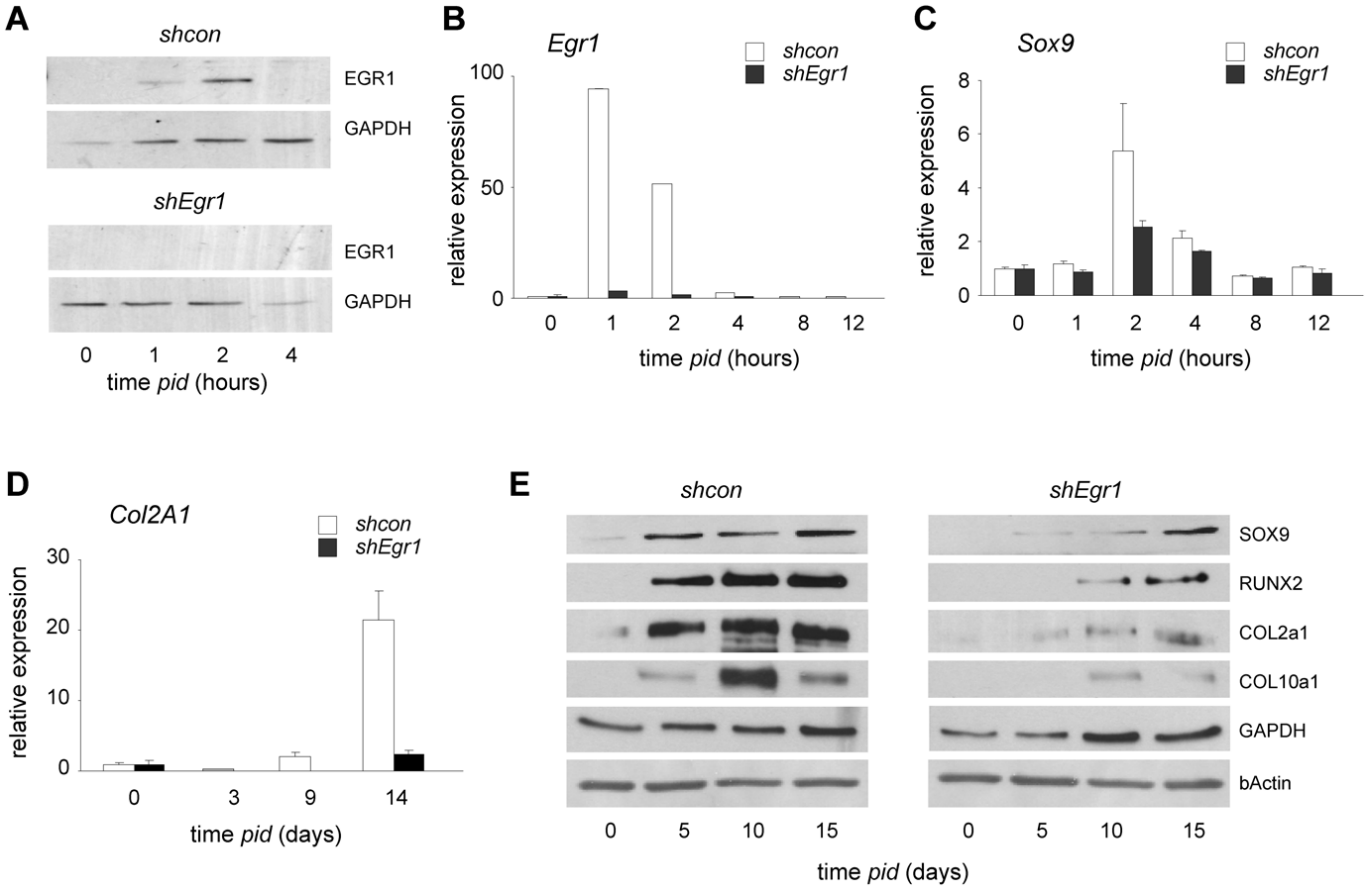
EGR1 depletion reduces chondrogenic differentiation. (A) EGR1-protein expression (protein) in ATDC5 cells stably expressing control short hairpin sequences (*shcon*) (upper panel); absent EGR1 in cells expressing *shEgr1* vectors (lower panel) at 0, 1, 2 and 4 hours pid. GAPDH is used as loading control. Samples corresponding to control and experiment (*shcon, shEgr1*) were loaded on the same gel to enable direct quantitative comparison (corresponding sections are shown separately; representative experiment shown. Selection pressure on *shRNA* expression was maintained for the duration of the experiments. (B–D) Reduced *Egr1* (B), *Sox9* (C) and *Col2a1* (D) expression (mRNA) in ATDC5 *shEgr1* compared to *shcon cultures*; standard error is based on three independent, parallel experiments; expression was normalized to *cyclophilin A*. (E) Reduced chondrogenic marker protein expression in ATDC5 cells stably expressing *shEgr1*. Samples corresponding to control and experiment (*shcon, shEgr1*) were loaded on the same gel to enable direct quantitative comparison (corresponding sections are shown separately; representative experiment shown).

To firmly establish a role for EGR1 in chondrogenesis, we studied chondrogenic marker gene expression in the context of RNA-interference mediated knock-down (KD) of Egr1. An *shRNA* vector targeting murine *Egr1* mRNA (*shEgr1*) was designed based on criteria previously published [43]. EGR1 protein expression in response to differentiation stimuli closely paralleled the observed changes in *Egr1* mRNA levels: whereas absent at t = 0, EGR1 protein was first detected at 1 hour *pid*, peaked at 2 hours and rapidly returned to control levels thereafter (Figure 3A). Expression analysis at both mRNA level and protein level showed that the retroviral *shEgr1* vector efficiently targets *Egr1* expression (Figure 3A,B). Both *Sox9* and *Runx2* displayed a multi-phasic mRNA expression profile throughout differentiation (Figure S1A; S2A,B). Relevantly, EGR1 protein depletion blunted early *Sox9* expression (Figure 3C; Figure S2C); *Runx2* mRNA expression was not maintained beyond 16 hours *pid* (Figure S2C). Both SOX9 and RUNX2 protein failed to be induced at early time points in differentiation (Figure 3E). Expression of genes encoding the ECM proteins Collagen type II (COL2A1) and AGC1, is typically activated 7–10 days *pid* in reference cultures; RUNX2 induced Collagen type X (*Col10a1*) expression coincided with hypertrophic differentiation [44]; Consistent with reduced chondrogenic capacity; *shEgr1* significantly reduced *Col2a1 expression* at the mRNA and protein levels (Figure 3D,E); COL10A1 protein levels were severely reduced in *shEgr1* cultures (Figure 3E). Loss of gene function often activates redundant mechanisms in important biological processes and may delay differentiation *in vitro*. We therefore studied the effect of EGR1 depletion on expression of the mammalian *Egr1* homologs *Egr2*, *3* and *4* and traced chondrogenic marker expression at advanced time points (*i.e.* beyond 2 weeks). In the absence of EGR1 *Egr2* and *Egr4* mRNA induction exceeded that of the control situation (Figure S6A); in contrast, *Egr3* induction and expression were not affected by EGR1 deficiency. Quantitative expression analysis of the chondrogenic markers *Sox9*, *Agc1*, *Col2A1* and *Col10A1* showed a delayed (5–10 days) differentiation response (Figure S6B); in light of other phenotypic changes in these cells (see below) it is currently not possible to assess whether the observed late marker gene expression reflects functional chondrogenic development.

Our combined findings demonstrate that activation of *Egr1* expression early in differentiation directly activates transcription of key chondrogenic regulators. In addition, H3K4me3-marked promoters are permissive to EGR1 binding, whereas local H3K27-trimethylation inversely correlates with EGR1/chromatin binding.

### Loss of EGR1 elicits replication-associated DNA damage and blocks hyper-proliferation

**Figure 4:**
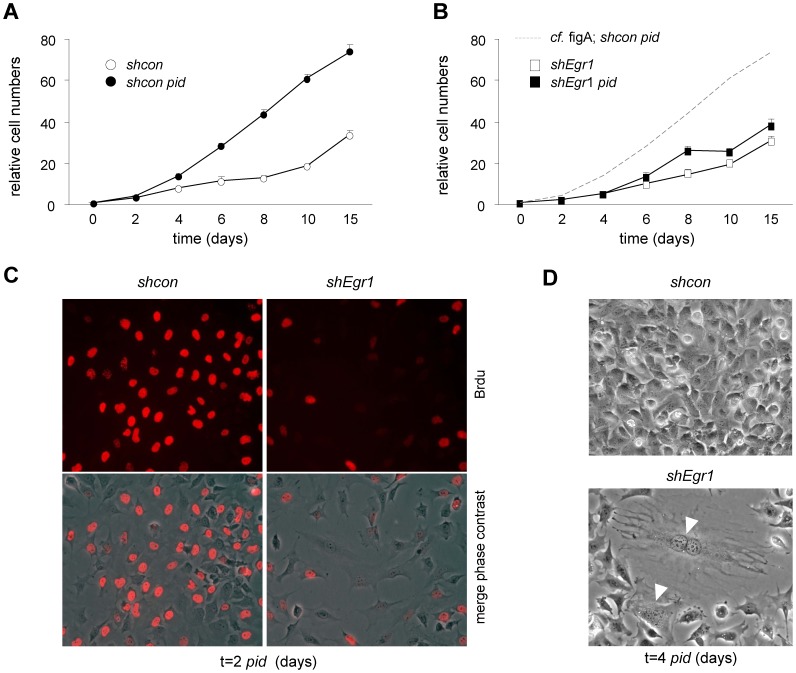
Loss of EGR1 elicits replication stress-induced senescence-like cell cycle arrest. (A,B) ATDC5 proliferation determined by Crystal violet incorporation. *Shcon* cells (A) show hyper-proliferation under differentiation conditions (*pid*); hyper-proliferation is blocked in *shEgr1* cultures (B). (C) Reduced replication (*de novo* DNA synthesis) and proliferation of *shEgr1* cells reflected by decreased BrdU -incorporation; IC detection of BrdU. (D) Detection of large flat cell morphology in ATDC5 cultures stably expressing *shEgr1* at 4 days *pid*; arrow heads indicate large polyploid cells; phase-contrast images.

**Figure 5:**
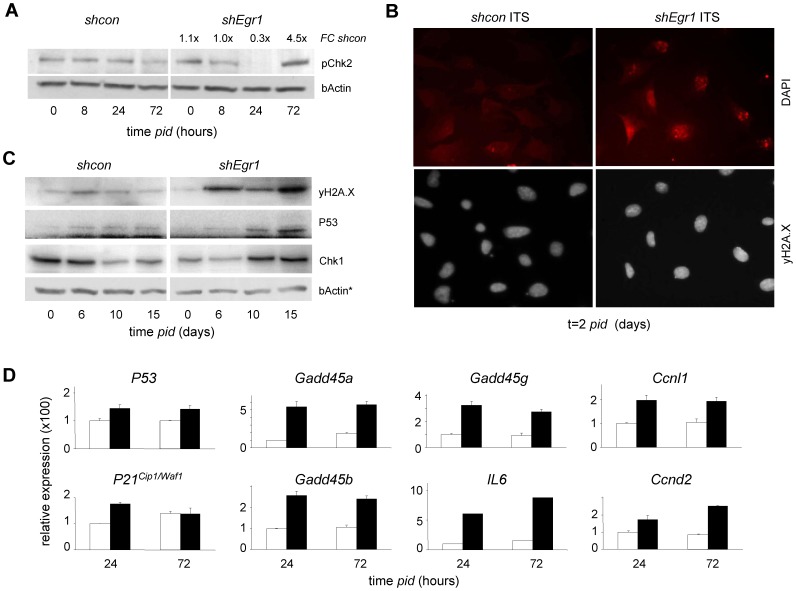
EGR1-LOF elicits DNA damage in hyper-proliferating chondrocytes. (A) Immunoblot (IB) detection of enhanced phospho-CHK2 (pCHK2) in *shEgr1* cultures early in chondrogenesis; FC *shcon*/*shEgr1*: 1,1 (t = 0), 1.0 (t = 8), 0.5 (t = 24), 4.5 (t = 72 days *pid*). (B) Detection of early DNA damage by IC analysis of γH2A.X (upper panels) in ATDC5 *shEgr1* cultures at 2 days *pid*; DAPI counterstaining by DAPI (lower panels). (C) IB detection of proteins related to DNA damage responses and cell-cycle arrest in ATDC5 cells stably expressing *shEgr1*; bActin loading control (* as in Figure 6B). Samples corresponding to control and experiment (*shcon, shEgr1*) were loaded on the same gel to enable direct quantitative comparison (corresponding sections are shown separately; representative experiment shown). (D) Comparative mRNA expression analysis of genes involved in cellular senescence, DNA damage response and stress signalling in *shcon* ATDC5 cells and cells stably expressing *shEgr1* throughout chondrogenesis (arbitrary expression units).

We next studied ATDC5 differentiation-induced proliferation as a function of EGR1. An important early feature of chondrogenesis is the capacity of progenitors to rapidly proliferate [28]. The proliferation rate of control cells is approximately 3 fold increased from 1 day *pid* onward as compared to cells under non-differentiating culture conditions (Figure 4A). Differentiating cells typically overgrow each other and form dense cellular nodules, *i.e.* focal points of chondrogenesis (Figure S7) [28], [29]. In our experimental setting the proliferative burst followed the transient EGR1 upregulation (*cf.* Figure 1B), indicating a potential requirement for EGR1 for enhanced cell division. In agreement with this idea, EGR1 deficiency completely abrogated the ability of ATDC5 cells to hyper-proliferate and cell proliferation in subconfluent cultures was strongly reduced (Figure 4B; Figure S7). The negative effect of *shEgr1* on differentiation-induced proliferation was independently confirmed using immunocytochemical (IC) detection of incorporation of the nucleotide-analog BrdU as a read-out (Figure 4C). The hyper-proliferation block was accompanied by dramatic morphological changes: EGR1 deficient cells displayed a ‘large flat cell’ phenotype and polyploidy (Figure 4D). As replication arrest was often associated with DNA damage, we examined *shErg1* cultures for evidence of DNA damage response (DDR). Activation of DDR was confirmed by detection of phosphorylated histone variant H2A.X (γH2A.X) and phospho-CHK2 (pCHK2): in control cells CHK2 was phosphorylated at a basal level and slightly reduced during hyper-proliferation, differentiation in the absence of EGR1 induced a substantial increase in pCHK2 at day 3 (4.5x higher compared to control cells; Figure 5A). Consistent with this, IC detection of γH2A.X confirmed DNA damage in *shEgr1* cells under differentiating conditions (Figure 5B). Upregulation of several other cell stress protein and mRNA markers suggested that DDR (CHK1, P53; Figure 5C) and cell stress response pathways were activated and sustained (*P53, P21^CIP1/WAF1^, Gadd45a,b,g, Ccnl1,d2*; Figure 5D). To map chondrogenic processes regulated by EGR1, genes identified in a genome-wide blast for EGR1 binding sites were analysed using GenMAPP; this yielded a number of overrepresented processes known to be involved in chondrogenesis and differentiation in general (Table S1); Comparison of pathway deregulation between sh*Erg1* and *shcon* cultures showed that multiple relevant pathways (*i.a.* differentiation, cell cycle regulation, senescence, metabolism, inflammatory responses) were upset by loss of EGR1 (Figure S8); relevantly, these processes significantly overlap with the above pathway analysis (Table S1). Taken together, this data clearly identifies EGR1 as an essential regulator protein for hyper-proliferation in the context of chondrogenesis and shows that loss of EGR1 results in defective hyper-proliferation.

### EGR1 controls early epigenomic remodeling and intersects with PRC function

**Figure 6:**
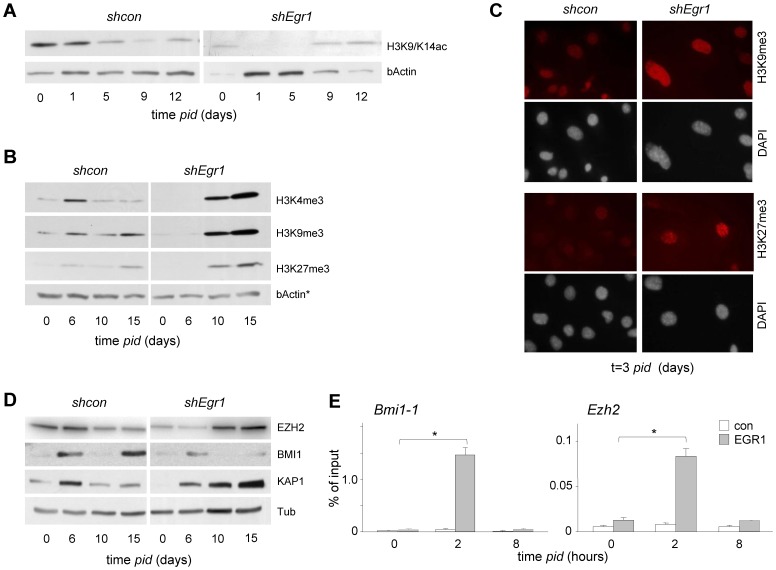
Loss of EGR1 affects chondrogenic histone modification and epigenetic modifier expression. (A, B) IB analysis of histone modifications ATDC5 *shcon* and *shEgr1* cultures as a function of chondrogenic differentiation time (as indicated): reduced histone acetylation (A) and abnormal histone trimethylation (B); bActin: loading control (* as in Figure 5B). (C) IC Detection of enhanced H3K9me3 staining (upper panels) and H3K27me3 staining in ATDC5 large flat *shEgr1* cells at 3 days *pid*; DAPI counterstaining by DAPI (lower panels). (D) IC detection of abnormal epigenetic regulator protein expression (BMI1, EZH2, KAP1) as a function of differentiation time; Tubulin loading control. Samples corresponding to control and experiment (Figures A, B, D; *shcon, shEgr1*) were loaded on the same gel to enable direct quantitative comparison (corresponding sections are shown separately; representative experiments shown). (E) EGR1 enrichment on BMI1 and EZH2 promoters at 0, 2 and 8 hours *pid*. *: P values (EGR1/chromatin enrichment at t = 2 vs t = 0): 0.045 and 0.05.

As EGR1 controls transcriptional reprogramming and hyper-proliferation, both DNA-templated processes associated with epigenomic remodeling, we studied global dynamic epigenomic changes throughout chondrogenesis as a function of EGR1. Histone acetylation (H3K9/K14ac), trimethylation at lysine 27 (H3K27me3), lysine 4 (H3K4me3) and lysine 9 (H3K9me3) all underwent dynamic changes throughout chondrogenesis: all trimethyl marks peaked during hyper-proliferation (6 days *pid*); following a reduction at 10 days *pid*, the repressive trimethylmarks (H3K9/K27) subsequently increased toward hypertrophy (Figure 6A,B). In contrast, histone acetylation was especially high in *shcon* chromatin at the initial stages of chondrogenesis, and gradually declined over time (Figure 6A). Remarkably, all epigenomic changes were substantially perturbed in *shEgr1* cultures. Initial global acetylation levels appear low in *shEgr1* cultures. Consistent with a role for EGR1 in recruiting HAT (histone acetyl transferase) activity, *shEgr1* cells also displayed a striking inability to induce and/or maintain acetylation levels in response to differentiation stimuli (Figure 6A). Trimethylation at histone H3K4, H3K9 and H3K27 appeared very low at t = 0 (Figure 6B); it is conceivable that absence of EGR1 affects HMT (histone methyl transferase) expression directly or indirectly, as observed for EZH2 (see below). Global trimethylation levels increased in EGR1-KD to levels much higher than observed in control cultures at t = 10 and t = 15, time points at which (based on *Col10A1* expression; *cf* Figure 3B) cells would normally undergo hypertrophy (Figure 6B). IC analysis confirmed enhanced H3K9me3 and H3K27me3 in nuclei of large flat cells (Figure 6C). We also examined the effect of EGR1 ablation on a number of H3K27me3 and K9me3 associated proteins. A substantial increase of KAP1/TIF1B expression, a factor associated with HP1/H3K9me3, correlated well with the observed increase in H3K9me3 levels at late time points in *shEgr1* cultures (Figure 6B–D) [45], [46]. We next focused on expression of BMI1 (a E3-Ubiquitin ligase for H2A) and EZH2 (a H3K27me3 HMT), representing PRC1 and PRC2 factors, respectively. Under normal conditions, the EZH2 protein level increased slightly in the context of early (t = 0/6) chondrogenic signaling. In sharp contrast, EZH2 levels were abnormally low at early time points and in *shEgr1* cultures yet showed a remarkable upregulation at later time points (Figure 6D; Figure S9A). Conversely, EZH2 expression was substantially enhanced in *shEgr1* cells from 10 days onward (Figure 6D); the abnormally high EZH2 level correlated well with the enhanced H3K27me3 detection (Figure 6B,C). BMI1 showed a biphasic induction profile at the protein level: it increases during hyper-proliferation in normal chondrogenic cultures and again late in differentiation (Figure 6D). EGR1 deficient cultures failed to induce BMI1 to the same extent as control cultures (Figure 6D; Figure S9A). The H3K4me3-enrichment and lack of H3K27me3, suggested that the *Ezh2* and the *Bmi1* locus were not embedded in transcriptionally repressive chromatin in ATDC5 cells (Figure S9B,C). The presence of multiple predicted EGR family member consensus binding sites in the Bmi1 promoter, and a more than 50 fold increased EGR1 occupation at the *Bmi1* promoter were consistent with a regulatory role for EGR1 in *Bmi1* expression (Figure 6E); the *Ezh2* promoter was significantly enriched, but to a lesser extent than *Bmi1* promoter (±4 fold). Although these findings demonstrate direct binding of EGR1 to the respective promoters, they do not rule out a requirement for additional regulatory factors for transcription. A substantial increase of KAP1/TIF1B expression, a factor associated with HP1/H3K9me3, correlates well with the observed increase in H3K9me3 levels at late time points in *shEgr1* cultures (Figure 6B–D) [45], [46].

**Figure 7:**
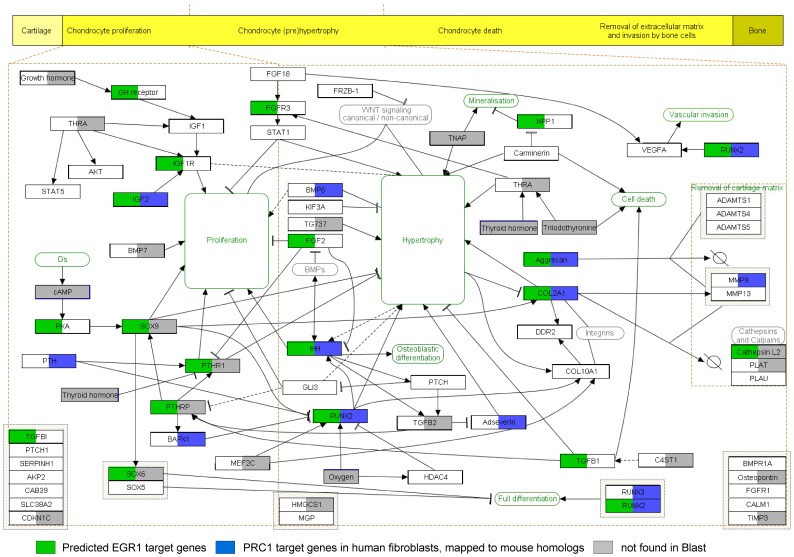
Chondrogenesis gene network. Enchondral ossification pathway analysis for predicted EGR1 targets (green) and published PRC1 targets (blue). A genome-wide blast search was performed for EGR1 consensus binding sequences; the search was confined to GCGG/TGGGCG motifs and its reverse complementary sequence (in sense and anti-sense orientation). Genes containing predicted binding-sites were mapped using pathway analysis tools (Pathvisio with Wikipathways content; see Methods section).

To chart potential target genes for EGR1 and to build a chondrogenic gene interaction network, we combined published data, with a mouse genome-wide *in silico* screen for putative EGR1 binding sites and software tools to predict promoter binding sites for numerous transcription factors (see: Methods section). The resulting network also depicts known Polycomb targets in cells of mesenchymal origin and shows sites of potential transcriptional co-regulation by EGR1 and PRC (Figure 7). Taken together, the above data demonstrates that EGR1 is crucial for initiation of genome-wide epigenetic reprogramming and that PRC and EGR1 are functionally linked in chondrogenesis.

## Discussion

We here report for the first time that many IEGs are rapidly induced in the context of a well-established *in vitro* model for chondrogenesis. We show that EGR1 is highly induced in response to differentiation stimuli. RNA-interference mediated knock-down of EGR1 affects expression of key chondrogenic regulatory genes, like *Sox9* and *Runx2*, and shows that EGR1 controls relevant chondrogenic pathways. Lack of EGR1 blocks early differentiation-induced hyper-proliferation and results in a number of cellular responses characteristic for cell stress-induced senescence. Loss of EGR1 affects early and late global chondrogenic epigenetic programming. Finally, our analyses reveal that EGR1 intersects with PRC function at the levels of PRC-gene transcription and obstructed EGR1/chromatin binding of promoters enriched for H3K27me3.

### EGR1 in chondrogenesis

Immediate early gene (IEG) induction denotes the first line of cellular responses to environmental and intrinsic stimuli, including growth factors, cytokines, differentiation signals and DNA-damaging agents. EGR1 has been functionally implicated as a mediator of inflammatory responses and is induced by the NFκB pathway in various experimental settings, including chondrocytes [47]–[52]. The etiological involvement of EGR1 in osteoarthritis (OA) is, however, unclear, as both increased and decreased expression of EGR1 has been reported in the context of OA-cartilage [53], [54]. Relevantly, we recently established that cell-autonomous activation of inflammatory pathways, i.e. NF-κB, is crucial for chondrogenesis [38], [55]. In addition, PRC control inflammatory responses [56] providing an additional potential function link between these cellular functions. Thus, although EGR1 has been implicated in several clinical aspects of cartilage physiology, its direct contribution to chondrogenesis was not known [17], [24], [54].

Published work on a conventional molecular genetic gene knock-out model for mouse *Egr1* (*NgfiA, Krox24, Zif268*) revealed no abnormalities in chondrogenic capacity [24]. A similar discrepancy between *in vivo* and *in vitro* findings was reported for EGR1s' role in retinal mircogliosis [57]. Given the occurrence of multiple gene orthologs in higher eukaryotes like the mouse, lack of phenotypic expression of a null- mutation gene is often attributed to activation of redundant mechanism. The *Egr* gene family consists of at least 4 members (*Egr1-4*). Indeed, EGR family members may regulate each other's expression, yet several studies provide evidence for distinctive functions for the individual EGR proteins: in the context of adipogenesis and T-cell activation, EGR1 and EGR2 have different roles [20], [58], [59]. In addition, individual *Egr* family knock-out mice display distinct memory related problems [60], [61]. Thus the effect of loss-of-function appears to be cell context dependent. Combining an *in vitro* system with acute RNAi-mediated knock-down also enabled us to isolate acute EGR1 dependent effects from reported redundant action of other EGR family members [62], [63]. The ATDC5-model we use here uniquely combines a number of relevant chondrogenic features: it reiterates the dynamic and strictly timed transcriptomic re-profiling observed during embryogenesis, and it incorporates a relevant proliferative increase typical of differentiating cells in the proliferative zone [28]–[33]. Although delayed marker gene expression suggests some late recovery of differentiation, we cannot formally rule out a late compensatory effect involving EGR1 dosage effects (for instance due to low viral copy number in a subset of cells) or involving other EGR paralogs. Definitive proof that EGR1 paralogs provide functional back-up in the context of EGR1 depletion requires combined loss of function models. Alternatively, the delayed marker expression could be the result of obligate transcriptional pre-programming. Although absence of an obvious chondrogenic phenotype *in vivo* suggests functional compensation for loss of EGR1 in chondrogenesis, this does not rule out important other functions for EGR1 in chondrocyte physiology and disease [47], [48], [64], [65]. However, the dramatic phenotypic changes, altered proliferative capacity and defective epigenetic remodelling in EGR1-depleted cells *in vitro* point to absence of functional compensation and uncover an important, cell autonomous role for EGR1 in early chondrogenesis.

### Defective epigenetic programming in absence of EGR1

The finding that EGR1 deficiency specifically blocks differentiation induced progenitor expansion strongly suggests that replication stress is involved in the hyper-proliferation block. Evidence for this is initially provided by the fact that chondrogenic differentiation induces ATDC5 cells to undergo an EGR1 dependent phase of hyper-replication. Secondly, EGR1 deficient cells accumulate DNA damage and activate a DDR as evidenced by phosphorylation of H2A.X, pCHK2, and upregulation of multiple DDR genes. Cell cycle exit and senescence are known to depend on activation of DDR [66]. We show here that DDR in EGR1-depleted cells coincides with strongly inhibited DNA replication and additional distinctive features suggesting that EGR1 deficient cells may be induced to undergo replicative senescence instead of differentiation: large flat cell morphology, polyploidy, expression of numerous senescence associated marker genes and involvement of relevant pathways. Of note, many of these pathways have been functionally linked to EGR1 [67], and are in concordance with our *in silico* analysis. We identified numerous cytokine signalling pathways as potential downstream targets of EGR1; relevantly, interleukins like IL6 have been implicated in senescence [68], [69]. Combined, this data strongly argues that EGR1 facilitates proliferative expansion in hyper-replicating chondrogenic progenitors.

The abnormal early global acetylation in the absence of EGR1 suggests that epigenomic reprogramming by EGR1 may serve to define concerted transcriptional or replication activity of gene-networks and support differentiation-specific changes in transcription and proliferation to guide cells through chondrogenesis. In keeping with such a coordinating role, EGR1 is known to induce, recruit and transactivate CBP/P300, the consequence of which is increased local HAT activity [70]–[72]. In analogy, the gene product of *cMyc*, one of the first IEGs identified, was found to augment global acetylation through both transcriptional regulation and recruitment of GCN5 [73]. cMYC was recently established to act as a general amplifier of gene expression in the context of development and differentiation, and in cells expressing abnormally high levels of MYC (*i.e.* in analogy to cMYC amplification in some cancers) [74], [75]. Our data suggest that by analogy, early EGR1 induction may fulfill a similar function during chondrogenesis. In the context of transcriptional regulation, among loci that harbor EGR1-consensus binding sites, we find gene promoters that: 1) appear to be directly induced by EGR1 (e.g. *Sox9*), 2) bind EGR1 but are not transcriptionally activated until later in chondrogenesis (e.g. *Sox4, Runx2*) or are not activated at all (*Hox* gene clusters), or 3) are blocked from EGR1 association by H3K27me3 (e.g. *Sox6*, *Agc1*) and become activated at later stages during chondrogenesis. Besides cooperation with transcriptional co-activators like NFATs and CBP/P330, EGR1 is known to interact with NAB1/2 and recruit the NuRD complex which represses transcription by bringing HDAC activity to target genes [76]–[79]. Many of these complex regulatory interactions and responses are likely to be cell context dependent. In line with this notion, several studies report cell type dependent responses of an EGR1 targeted reporter gene that was activated in neural cells, whereas in cartilage and heart it was suppressed [80], [81]. Thus, whether or not a locus is transcriptionally activated during chondrogenesis likely depends on the presence and/or recruitment of additional co-factors and/or epigenetic marks, besides EGR1.

### Integration of EGR1 and Polycomb Repressive Complex function

We and others have recently shown that Polycomb function is regulated by post-translational modifications suggesting a link between cell signaling and epigenetic re-programming involving PRC [14]. Interestingly, we here observe functional interaction of EGR1 with PRC function at several levels. Firstly, loss of EGR1 affects, directly or indirectly, expression of two important PRC members, EZH2 and BMI1. The Polycomb Repressive Complexes PRC2 and PRC1 are both functionally connected to the H3K27me3 mark. EZH2 is a histone lysine methyl transferase (HKMT) in Polycomb Repressive Complex 2 (PRC2), which trimethylates histone H3 at lysine 27 [82]. BMI1 is part of PRC1, which is recruited to H3K27me3 marks and believed to maintain transcriptionally repressed states [26], [83], [84]. As such, PRCs sustain lineage-commitment in the context of development [15]. Both proteins have been associated with cell cycle regulation: *Ezh2* is a direct target of E2F1 and Bmi1 controls expression of P16/INK4A and P14^ARF^, both encoded by the *CDKN2/INK4A* locus [85], [86]. Repression of this locus by PRC1 is crucial in early development [87]. Secondly, comparative analysis of published PRC targets and a genome-wide scan for consensus EGR1 binding sites yielded potential common targets, such as *Sox9, Runx2 and Ig2* and suggests coordinated regulation of gene expression in chondrogenesis (Figure 7). PRC1 mutation often results in defective body patterning reflected in abnormal skeletogenesis, due to loss of expression boundaries in the *Hox* gene clusters [40]. Based on our current analyses we cannot formally rule out a function for EGR factors in skeletogenesis, we present evidence that EGR1 and PRC functionally interact during chondrogenesis. We observe a close correlation between the presence of PRC-repressive H3K27me3 marks and lack of EGR1 enrichment at promoters of chondrogenic marker genes and at the *HoxA* cluster, suggesting that H3K27me3-decorated chromatin prevents EGR1 from accessing promoters. Interestingly, by analogy, H3K27me3 prevents cMYC from interacting with its genomic targets [88] [75]. Finally, we observe that EZH2 and BMI1 expression is abnormal in EGR1 deficient cells; since PRC1 deficiency is known to affect skeletogenesis in molecular genetic mouse models [16], at least part of the chondrogenic defect in EGR1 depleted cells may be explained by functional interaction with PRC.

At late time points, elevated H3K27me3 correlates with increased *Ezh2* expression. EZH2 levels were reported to decrease in the context of replicative senescence [89]. It is conceivable, that the formation of trimethylated chromatin represents (part of) a protective response against abnormal differentiation, transformation and/or unscheduled cell death. Also H3K9me3 is a hallmark of repressed chromatin. H3K9me3 and HP1 accumulation at distinct nuclear foci has been associated with replication stress induced senescence [90]. Increased accumulation of H3K9me3 at so-called senescence associated heterochromatin foci (SAHF) is the consequence of subnuclear redistribution rather than increased global H3K9me3 levels [91]. We identified several indicators of a senescence-like response in *shEgr*1 cultures that had been induced to differentiate. Our findings also show a global H3K9me3 increase at later time points (10, 15 days). Elevation of H3K9me3 is preceded by increased KAP1 expression; KAP1 is known to increase heterochromatin formation through recruitment of SETDB1 and CDH3 [45], [92]. KAP1 is specifically required to resolve DNA damage in heterochromatin, downstream of ATM signaling [93]. As KAP1 and HP1 physically interact [46], the elevated global H3K9me3 levels may point to increased heterochromatinization. DNA damage in senescent cells was reported to persist for weeks on end [94]; as such H3K9me3 and KAP1 may provide a means to prevent damaged DNA from replicating. It is currently only possible to speculate about the biological relevance of the co-regulation of seemingly conflicting trimethyl levels on H3K4, H3K9 and H3K27 under normal and stressed conditions. Apart from transcriptional regulation, each mark is associated with distinctive other processes. Increased H3K4me3 and H3K27me3 have both been associated with other cellular stress responses: we and others have observed increased H3K4me3 and H3K27me3 in response to conditions that evoke replication stress (*unpublished results*). In addition, H3K4me3 was identified as a crucial determinant of RAG2 mediated V(D)J recombination in B and T cells, and was linked to DDR induced cellular responses via ING family proteins [95], [96]. Such observations suggest an involvement of global trimethyl marking in cellular processes other than local regulation of transcription. It will be of considerable interest to globally map K4, K9 and K27 trimethyl, acetyl and additional epigenetic marking in respect to EGR1 binding and to correlate these marks to DNA-templated activity in the ATDC5 *shEgr1* system.

All DNA-templated processes, including transcription, DNA replication and DNA damage repair, are controlled by epigenetic mechanisms: access to DNA is controlled by covalent modification of histone proteins and chromatin structure remodeling [97]. The ATDC5 genome has to cope with significant transcriptome reprogramming as well as enhanced DNA replication during chondrogenesis. Both processes have to be coordinated to prevent cell stress. We demonstrate here that loss of EGR1 affects chondrogenic differentiation (*i.e.* transcription) as well as early proliferation. Hyper-proliferation sets in at approximately 24 hours into chondrogenesis; given the immediate early peak-response in EGR1 synthesis, the early rise in EGR1/chromatin occupation and its rapid degradation, it is unlikely that EGR1 is directly physically responsible for this coordination. Instead, these observations suggest that EGR1 helps to generate the conditions under which these DNA-templated processes can co-occur. The global distribution of EGR1 binding sites may point to a more general task in epigenomic reprogramming, not exclusively linked to transcription. By analogy, recent studies on genomic distribution of transcription factor (TF) binding sites (PPARy, ERa) identified up to half of such binding sites either in intragenic regions (introns) or at distant locations (>25 kb away from the nearest gene), and may suggest additional epigenomic roles besides TF binding in gene promoters [98]–[100]. It is tempting to propose a role for IEGs in early epigenomic pre-programming, such that ensuing processes (concurrent transcription and replication) are facilitated in the context of development.

## Conclusion

Although the importance of epigenetic regulatory mechanisms in differentiation is evident, exact knowledge on how cells communicate environmental changes to chromatin and how global epigenomic remodeling accompanies differentiation is lacking. Here, we report a dual function for the transcription factor EGR1 in activating a lineage specific transcriptional re-programming, as well as rapid proliferative progenitor expansion. The inappropriate chromatin reprogramming in the absence of EGR1 strongly support an important task for EGR1 in early epigenomic remodeling during chondrogenesis and pave the way for further studies on gene-environment interactions in development and cancer.

## Methods

### Cell culturing

The murine ATDC5 cell line was established and first reported on by Atsumi et al. [28]. ATDC5 cells were cultured at 37°C, 5% CO2, 100% humidity in DMEM/F-12 supplemented with 5% fetal calf serum (FCS), antibiotics (100 units/ml penicillin and 100 µg/ml streptomycin), 200 mM L-glutamine on tissue culture plates (Greiner Bio-One). For differentiation experiments cells were seeded at 6400 cells/cm^2^ and were allowed to attach overnight. Growth medium was changed for differentiation medium, which includes ITS (10 µg/ml insulin, 10 µg/ml transferrin and 3×10^−8^M sodium selenite) and puromycin (2 µg/ml). Differentiation medium was replaced every two days.

### Retroviral transduction

Retroviral systems and Phoenix helper-free retrovirus producer cell lines were used as published before [101], [102]. Ecotropic retroviral supernatants were produced by transfection of producer cells with calcium-phosphate precipitation; 24–48 hours post-transfection, the supernatant was harvested, filtered and used for infection of ATDC5 cells in presence of 4 µg/ml polybrene. Cells were incubated for 12 hours and then allowed to recover for 24 hours on fresh medium before selection pressure was applied. Infected cells were selected with puromycin (8 µg/ml) for 72 hours, before experiments were initiated; at the onset of experiments the puromycin concentration was lowered to 2 µg/ml for the duration of the experiment. Short-hairpin (sh)RNA target sequence for murine *Egr1*: 5′- ACAAAGTAACCTGTTTGGC-3′. Short hairpin sequence targeting *shGFP* (control) was used as reference sh-sequence [103].

### Affymetrix gene arrays & bioinformatics

Multiple consensus binding sequences for EGR family members exist [104]–[106]. Genomic scans were confined to GCGG/TGGGCG motifs and its reverse complementary sequence, both in sense and antisense orientation. Genes involved in chondrogenesis that carry predicted EGR1 consensus binding sites and H3K27me3 decoration (*cf.* Figure 7) [1], [9], [26] were mapped using pathway analysis tools (Pathvisio with Wikipathways content) [107], [108]. *In silico* promoter analyses for potential EGR1 binding sites was carried out using GENOMATIX software (http://www.genomatix.de).

For gene expression arrays, three independent replicate RNA samples taken at each time point during differentiation (0, 2, 4, 8, 16. 24 and 72 hours) from *shcon* and *shEgr1* cultures. Total RNA was isolated using the RNeasy kit (Qiagen) according to the manufacturers' protocol. The isolated RNA samples were processed by ServiceXS BV (Leiden, Netherlands) according to Affymetrix (Santa Clara, CA) protocols. In brief, RNA concentration was measured by absorbency at 260 nm using the Nanodrop (Nanodrop Technologies, Wilmington, DE, U.S.A), and RNA quality and integrity was verified by using the RNA 6000 Nano assay on the Agilent 2100 Bioanalyzer (Agilent Technologies, Palo Alto, CA). For each sample 2 ug high quality total RNA was labeled using the Affymetrix Eukaryotic One-Cycle Target Labeling and Control reagents to generate Biotin-labeled cRNA. The quality of the cRNA was verified using the Agilent 2100 bioanalyzer and the concentration was measured using the Nanodrop (Nanodrop Technologies, Wilmington, DE, U.S.A). Labeled cRNA was used for hybridization to 42 Affymetrix Mouse GeneChip arrays (NuGO_Mm1a520177). After an automated process of washing and staining, absolute values of expression were calculated from the scanned array using the Affymetrix GCOS software. Data preprocessing and analysis was conducted based on scripts from ArrayAnalysis.org using R2.7.1 and Bioconductor libraries (http://www.R-project.org) [109]. Data were 2log transformed and normalized (gcRMA) [110]. Probe annotations were updated using the Ensembl based Brainarray annotation file [111]. Statistics computation for each reporter on the arrays included: the average (2logged) expression of each group, the logratio (2log fold change) between the two groups and the t- and p-value of a Student t-test, all for each time point. Subsets of reporters fulfilling specific criteria were selected for various analyses, where generally cut-offs on p-value, logratio, and minimum expression (p<0.05, absolute FC (fold change) ≥2, expression >2log(100) in either group, at any time point) were imposed and controls removed from the data set. For pathway Z score analysis, expression at any given time point was compared between the respective genotype and referenced to t = 0; overall *shEgr1*/t = 0 expression signal was normalized against *shcon*/t = 0. GenMAPP was used for biological process annotation [112]. Overrepresentation of biological processes was determined using PathVisio (http://www.pathvisio.org) using pathways available through WikiPathways (www.wikipathways.org) [107], [108]. Microarray data have been deposited in the ArrayExpress (www.ebi.ac.uk/arrayexpress/) databse with series accession code E-MTAB-1464.

### RNA isolation, cDNA synthesis, quantitative PCR analysis

For quantitative PCR (qPCR) analysis, total RNA from three independent parallel experiments was isolated using Tri-Reagent (Sigma) according to the manufacturers' protocol. Quantity and quality of the RNA were determined by 260/280 nm and 260/230 nm absorbance measurements, respectively, using the Nanodrop (Witec AG, Luzern, Switzerland). Total RNA (1 µg) for each sample/replicate was converted into first strand cDNA using the iScript™ cDNA synthesis kit (Bio-Rad, Herculus, CA, USA) according to the manufacturers' instructions. Gene expression was determined by real-time qPCR using the MyiQ™ Thermal Cycler (Bio-Rad) in combination with the IQ5 v2 software (Bio-Rad). qPCR was performed on 25 ng of cDNA using the qPCR iQ™ Custom SYBR® Green Supermix with fluorescein (Bio-Rad) and 300 nM primer in 96 well plates (Bio-Rad). For each primer pair a standard curve was generated with a serial dilution of a cDNA pool. qPCR data was analyzed according to the relative standard curve method. All values were normalized to *cyclophillin A*. The control conditions were used as a reference. Primer sets for the selected genes were developed with Primer Express version 2.0 (Applied Biosystems, Foster City, CA, USA) using default settings (see Table S2).

### Chromatin Immunoprecipitation (ChIP)

ChIPs were performed and analysed essentially as described previously [26]. Briefly, ATDC5 cells were fixed in 1% formaldehyde. Cross-linking was allowed to proceed for 10 min at room temperature and stopped by addition of glycine at a final concentration of 0.125 M, followed by an additional incubation for 5 min. Fixed cells were washed twice with PBS and harvested in SDS Buffer (50 mM Tris at pH 8.1, 0.5% SDS, 100 mM NaCl, 5 mM EDTA), supplemented with protease inhibitors (Aprotinin, Antipain and Leupeptin all at 5 µg/ml and 1 mM PMSF). Cells were pelleted by centrifugation, and suspended in IP Buffer (100 mM Tris at pH 8.6, 100 mM NaCl, 0.3% SDS, 1.7% Triton X-100 and 5 mM EDTA), containing protease inhibitors. Cells were disrupted by sonication, yielding genomic DNA fragments with a bulk size of 200–500 bp. For each immunoprecipitation, 1 ml of lysate was precleared by addition of 35 µl of blocked protein A beads (50% slurry protein A-Sepharose, Amersham; 0.5 mg/ml fatty acid-free BSA, Sigma; and 0.2 mg/ml herring sperm DNA in TE), followed by clarification by centrifugation. 10 µl aliquots of precleared suspension were reserved as input DNA and kept at 4°C. Samples were immunoprecipitated overnight at 4°C with antibodies for either HA as a negative control (sc-805; Santa Cruz), H3K27me3 (07-449; Upstate), anti H3K4me3 Poab (ab8580) (Abcam), anti EGR1 Poab sc-110 (Santa Cruz Biotechnology). Immune complexes were recovered by adding 40 µl of blocked protein A beads and incubated for 4 h at 4°C. Beads were washed three times in 1 mL of mixed micelle buffer (20 mM Tris at pH 8.1, 150 mM NaCl, 5 mM EDTA, 5% w/v sucrose, 1% Triton X-100, and 0.2% SDS), twice in 1 mL of Buffer 500 (50 mM HEPES at pH 7.5, 0.1% w/v deoxycholic acid, 1% Triton X-100, and 1 mM EDTA), twice in 1 mL of LiCl Detergent wash buffer (10 mM Tris at pH 8.0, 0.5% deoxycholic acid, 0.5% NP-40, 250 mM LiCl, and 1 mM EDTA), and once in 1 ml of TE. Immuno-complexes were eluted from beads in 250 µl elution buffer (1% SDS; and 0.1M NaHCO3) for 2 hrs at 65°C with continuous shaking at 1000 rpm, and after centrifugation supernatants were collected. 250 µl elution buffer was added to input DNA samples and these were processed in parallel with eluted samples. Crosslinks were reversed overnight at 65°C followed by a 2 hrs digestion with RNAse A at 37°C and 2 hrs proteinase K (0.2 µg/µl) at 55°C. DNA fragments were recovered using QIAquick PCR purification columns, according to manufacturers' instructions. Samples were eluted in 75 µl EB buffer and then further 1/5 diluted in TE buffer. The immunoprecipitated DNA was quantified by real-time PCR (see section above). For corresponding primer sequences see Table S2. Data presented are based on duplicate measurements; statistical significance was assessed by Welch's *t* test and indicated where applicable.

### Cell proliferation assays

ATDC5 cells were plated in 12-multiwell plates (Greiner Bio-one). At each time point cells were washed twice with phosphate-buffered saline, and fixed for 10 minutes with 3.7% formaldehyde at room temperature. Next, cells were rinsed 5 times with demiwater. Cells were stained with 0.1% Chrystal violet for 30 minutes or overnight, and washed 5 times with demiwater. Chrystal violet was extracted with 10% acetic acid and absorbance was measured spectrophotometrically at 590 nm (Benchmark, Biorad).

### Immunobotting (IB)

Cells were lysed in RIPA buffer containing 5 mM Benzamidine, 5 µg/ml Antipain, 5 µg/ml Leupeptin, 5 µg/ml Aprotinin, 1 mM Sodium Vanadate, 10 mM Sodium Fluoride, 10 mM Pyrophosphate, 10 mM β-glycerophosphate, 0.5 mM DTT and 1 mM PMSF. Lysates were subjected to 3 freeze-thaw cycles in liquid nitrogen, and to 3 cycles of sonication. After centrifugation for 10 minutes at 13.2 k rpm, protein concentration was determined using a BCA protein assay kit (Pierce). Equal amounts of protein were boiled in Laemmli buffer and loaded on 9–15% polyacrylamide gels. Proteins were transferred to polyvinylidene fluoride (PVDF) membranes. After blocking with 5% non-fat dry milk in PBS containing 0.1% Tween-20, membranes were incubated o/n at 4°C with the following antibodies: β-actin Mab (C4, 691001, MP Biomedicals), GAPDH, EZH2 Mab (BD43; courtesy D. Pasin Copenhagen, Denmark), BMI1 Mab (F6; courtesy M. vanLohiuzen; Amsterdam, The Netherlands), EGR1 Poab sc-110 (Santa Cruz Biotechnology), CHK1-DCS-310 (Abcam Ab22610), pCHK2 (Thr68) #2661 (Cell signaling), γH2A.X (Ser139) Poab #2577 (Cell signaling), P53 Mab (DAKO), Geminin (FL-209) Poab (Santa Cruz Biotechnology), H3K4me3 Poab (ab8580) (Abcam), H3K27me3 Poab, #07-449 (Upstate), Kap1 Poab A300-275A (Bethyl Laboratories, H3K9/14Ac Poab (#06-599, Upstate Biotechnology). After extensive washing, membranes were incubated with corresponding horseradish peroxidase conjugated secondary antibodies for 1 h at room temperature. Signals were detected using enhanced chemoluminescence.

### Immunocytochemistry (IC)

Briefly, cells were washed and fixed for 10–15 minutes in 100% methanol at −20°C and stored at 4°C in 70% ethanol or used directly for immunocytochemistry. Cells were permeabilized for 5–10 minutes in 0.2& triton-X in PBS. To prevent epitope loss in combination with acid treatment for BrdU detection, primary antisera were incubated for 1.5 hours at 37°C, 4–5x washed in 0.02% triton-X/PBS and fixed in 2% formaldehyde/PBS for 10 minutes at room temperature. Cells were washed with PBS and incubated for 20 minutes in 2.0N HCl at 37°C, followed by two rinses of 0.1 M sodium tetraborate solution, pH 8.5 for in total 2 minutes. Cells were than incubated with the primary antisera against BrdU, washed at indicated before and incubated with secondary fluorescently labeled antibodies. All antisera were diluted and incubated in 0.02% triton-X in PBS. Nuclei were counterstained with 4′-6-Diamidino-2-phenylindole (DAPI) and washed in 0.02% triton-X/PBS. The last wash step was in PBS, upon which cells were dehydrated: 1 minute in 70% ethanol, 2x 1 minute in 100% ethanol and air-dried. Cells were mounted in Vectashield (Vector Laboratories, Inc. Burlingame, CA) and analyzed using a NIKON TE200 Eclipse fluorescence microscope and photographed using a NIKON DXM1200 digital camera in combination with NIS Elements 3.0 Imaging software. The following antibodies were used: γH2A.X Mab JBW301 (Upstate), BrdU (BD biosciences) and H3K9me3 #07-442 (Upstate), H3K27me3 Poab, #07-449 (Upstate).

## Supporting Information

Figure S1 **Sox9 promoter contains putative EGR1 binding sites.** (A) Biphasic *Sox9* expression profile (relative expression in arbitrary units). (B) promoter analysis of chondrogenic promoters for EGR1 binding sites (black triangles; GENOMATIX-based approach; see Methods section); forward and reverse black arrows indicate primer locations for qPCR of immuno-precipitated chromatin. (C,D) EGR1 occupation (C) and H3K4me3 and H3K27me3-enrichment (D) at *Sox9* promoter at 0, 2 and 8 hours *pid*; control (con) ChIP experiments were carried out with a non-relevant haemagglutinin (HA) anti-serum. *: P value (EGR1/chromatin enrichment at t = 2 vs t = 0): 0.06.
(TIF)

Figure S2 **Expression profiles of chondrogenic marker genes.** (A,B) multi-phasic *Runx2* expression profile (A) and late expression of *Agc1* (B), as a function of normal chondrogenesis (relative expression in arbitrary units). (C) Comparative expression profiling of *Sox9*, *Runx2* and *Agc1* in *shcon* and *shEgr1* ATDC5 cultures (expression array analysis, arbitrary expression units).
(TIF)

Figure S3 **EGR1 binding is blocked by H3K27me3 at the** ***Sox6*** **promoter.** (A) Late expression of *Sox6* during normal chondrogenesis (relative expression in arbitrary units). (B) Comparative expression profiling of *Sox6* in *shcon* and *shEgr1* ATDC5 cultures (expression array analysis, arbitrary expression units). (C,D) EGR1 occupation (C) and H3K4me3 and H3K27me3-enrichment (D) at *Sox6* promoter at 0, 2 and 8 hours *pid*; control (con) ChIP experiments were carried out with a non-relevant haemagglutinin (HA) anti-serum. P value (EGR1/chromatin enrichment at t = 2 vs t = 0): 0.35.
(TIF)

Figure S4 **EGR1 binding at the** ***Sox4*** **promoter does not activate transcription.** (A) Expression profile of *Sox4* in the context of normal chondrogenesis (relative expression in arbitrary units). (B) Comparative expression profiling of *Sox4* in *shcon* and *shEgr1* ATDC5 cultures (expression array analysis, arbitrary expression units). (C) EGR1 occupation at the *Sox4* promoter at 0, 2 and 8 hours *pid*. Control (con) ChIP experiments were carried out with a non-relevant haemagglutinin (HA) anti-serum. *: P value (EGR1/chromatin enrichment at t = 2 vs t = 0): 0.043.
(TIF)

Figure S5 **Hox gene expression in the presence and absence of EGR1.** mRNA expression analysis of all Hox clusters in ATDC5 cells stably expressing shcon or *shEgr1* throughout chondrogenesis; data are based on array analysis of three independent replicate RNA samples (normalization was done against *cyclophyllin A*).
(TIF)

Figure S6 **Delayed expression of chondrogenic marker genes in the absence of EGR1.** (A) Expression of Egr gene family members 2, 3 and 4 in the absence of EGR1; data are based on array analysis of three independent replicate RNA samples (normalization was done against *cyclophyllin A*). (B) qPCR analysis of delayed chondrogenic markers expression *Sox9*, *Agc1*, *Col2A1* and *Col10A1* in the absence of EGR1; standard error is based on three independent, parallel experiments; expression was normalized to *cyclophilin A*.
(TIF)

Figure S7 **Loss of EGR1 affects differentiation induced hyper-proliferation.** Morphological analysis of ATDC5 cultures stably expressing either shcon or *shEgr1* vectors. Note that at 3 days *pid* differences in cell density are detectable and large flat cells appear (arrow heads); *shEgr1* cultures do not reach super-confluence and do not form chondrogenic nodules (circles).
(TIF)

Figure S8 **Deregulation of pathway in EGR1 deficient cultures.** Pathway-heatmap of Z scores *shEgr1* versus *shcon*. Pathways with a Z score of minimally 1.9 at any given time point are depicted; inset shows key to heatmap-colors (yellow: more genes/pathway, dark red: less genes/pathway deregulated in *shEgr1* culture than expected on basis of random distribution) and histograms (percentage of genes deregulated in any given pathway).
(TIF)

Figure S9 **Reduced expression of PRC1 and PRC2-complex members BMI1 and EZH2 in** ***shEgr1*** **-cultures.** (A) Comparative mRNA expression of *Bmi1* and *Ezh2* in ATDC5 cells stably expressing *shcon* or *shEgr1* as a function of differentiation. (B) promoter analysis of chondrogenic promoters for EGR1 binding sites (black triangles; GENOMATIX-based approach; see: Methods section); forward and reverse black arrows indicate primer locations for qPCR of immuno-precipitated chromatin. (C) H3K4me3 and H3K27me3-enrichment at *Bmi1* and *Ezh2* promoters at 0, 2 and 8 hours *pid*; control (con) ChIP experiments were carried out with a non-relevant haemagglutinin (HA) anti-serum.
(TIF)

Table S1 **Putative pathway regulation by** ***in silico*** **mapping of EGR1 binding sites.**
(TIF)

Table S2 **Primers used for quantitative PCR.**
(TIF)
