# Supplementary figures and images for: The Immediate Early Gene Product EGR1 and Polycomb Group Proteins Interact in Epigenetic Programming during Chondrogenesis
Source: PLoS One. 2013 Mar 6;8(3):e58083. doi: 10.1371/journal.pone.0058083 (PMC3590300; doi:10.1371/journal.pone.0058083)

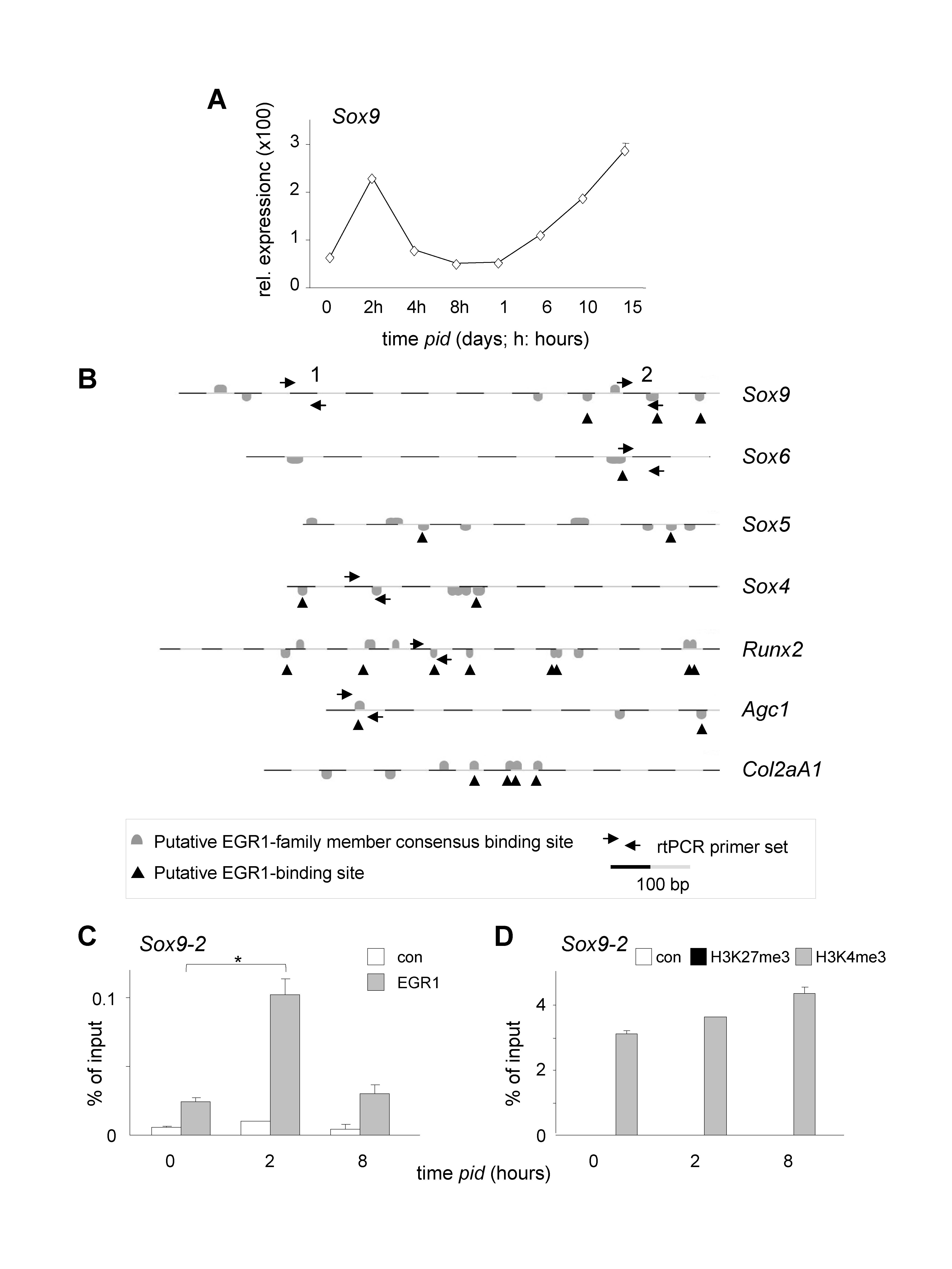

Supplement: Figure S1 — Sox9 promoter contains putative EGR1 binding sites. (A) Biphasic Sox9 expression profile (relative expression in arbitrary units). (B) promoter analysis of chondrogenic promoters for EGR1 binding sites (black triangles; GENOMATIX-based approach; see Methods section); forward and reverse black arrows indicate primer locations for qPCR of immuno-precipitated chromatin. (C,D) EGR1 occupation (C) and H3K4me3 and H3K27me3-enrichment (D) at Sox9 promoter at 0, 2 and 8 hours pid; control (con) ChIP experiments were carried out with a non-relevant haemagglutinin (HA) anti-serum. *: P value (EGR1/chromatin enrichment at t = 2 vs t = 0): 0.06. (TIF) [file pone.0058083.s001.tif]

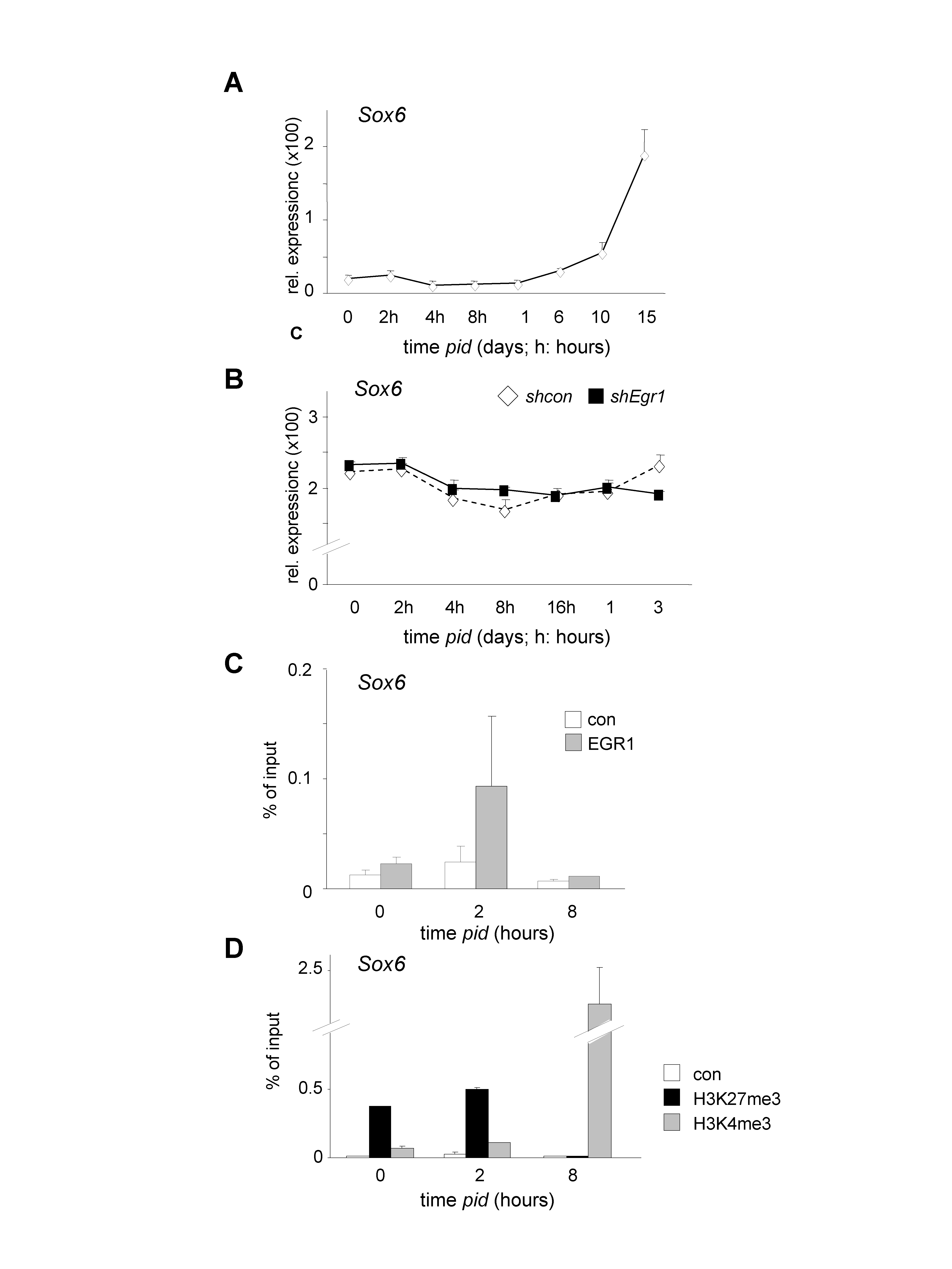

Supplement: Figure S3 — EGR1 binding is blocked by H3K27me3 at the Sox6 promoter. (A) Late expression of Sox6 during normal chondrogenesis (relative expression in arbitrary units). (B) Comparative expression profiling of Sox6 in shcon and shEgr1 ATDC5 cultures (expression array analysis, arbitrary expression units). (C,D) EGR1 occupation (C) and H3K4me3 and H3K27me3-enrichment (D) at Sox6 promoter at 0, 2 and 8 hours pid; control (con) ChIP experiments were carried out with a non-relevant haemagglutinin (HA) anti-serum. P value (EGR1/chromatin enrichment at t = 2 vs t = 0): 0.35. (TIF) [file pone.0058083.s003.tif]

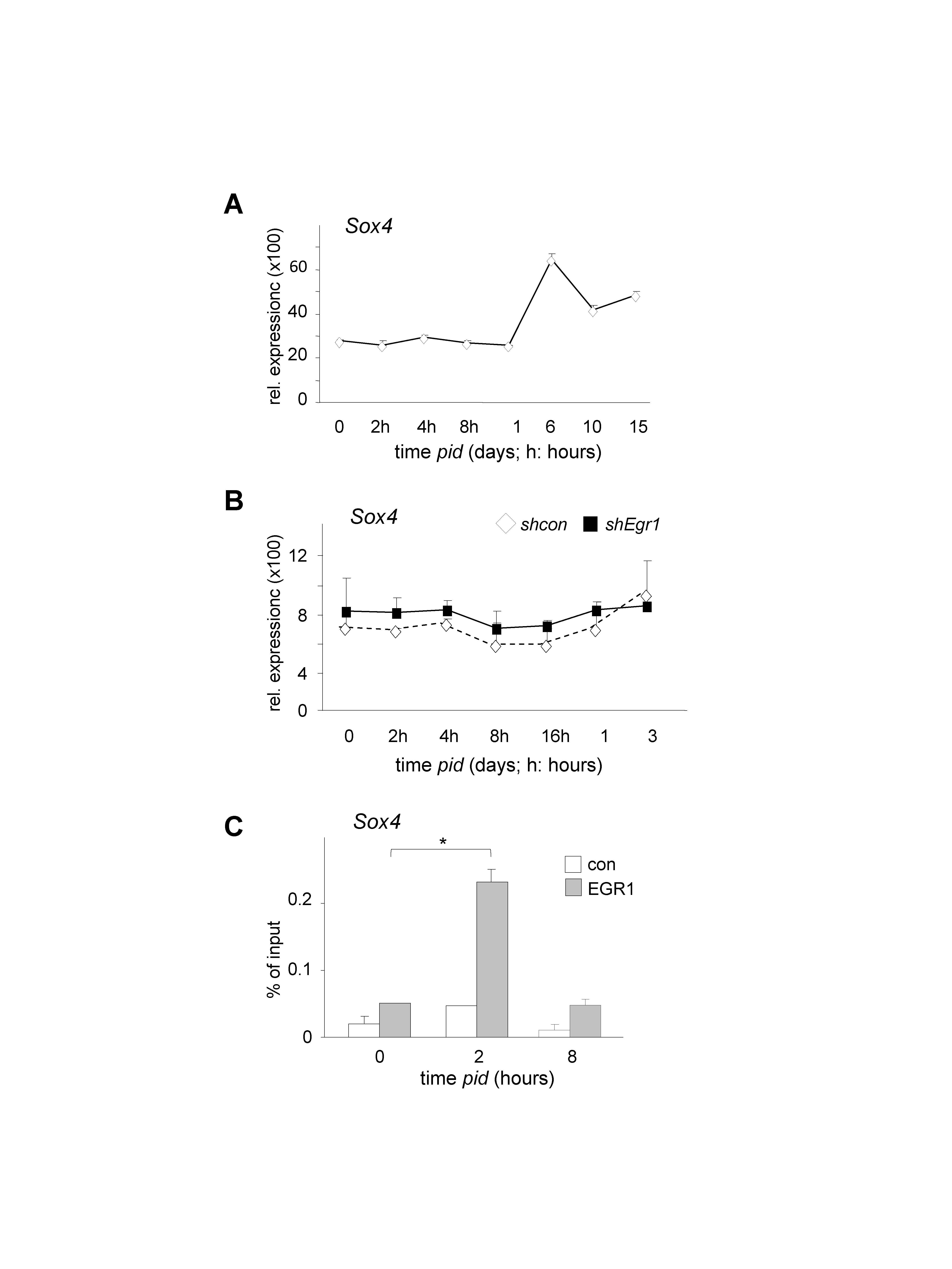

Supplement: Figure S4 — EGR1 binding at the Sox4 promoter does not activate transcription. (A) Expression profile of Sox4 in the context of normal chondrogenesis (relative expression in arbitrary units). (B) Comparative expression profiling of Sox4 in shcon and shEgr1 ATDC5 cultures (expression array analysis, arbitrary expression units). (C) EGR1 occupation at the Sox4 promoter at 0, 2 and 8 hours pid. Control (con) ChIP experiments were carried out with a non-relevant haemagglutinin (HA) anti-serum. *: P value (EGR1/chromatin enrichment at t = 2 vs t = 0): 0.043. (TIF) [file pone.0058083.s004.tif]

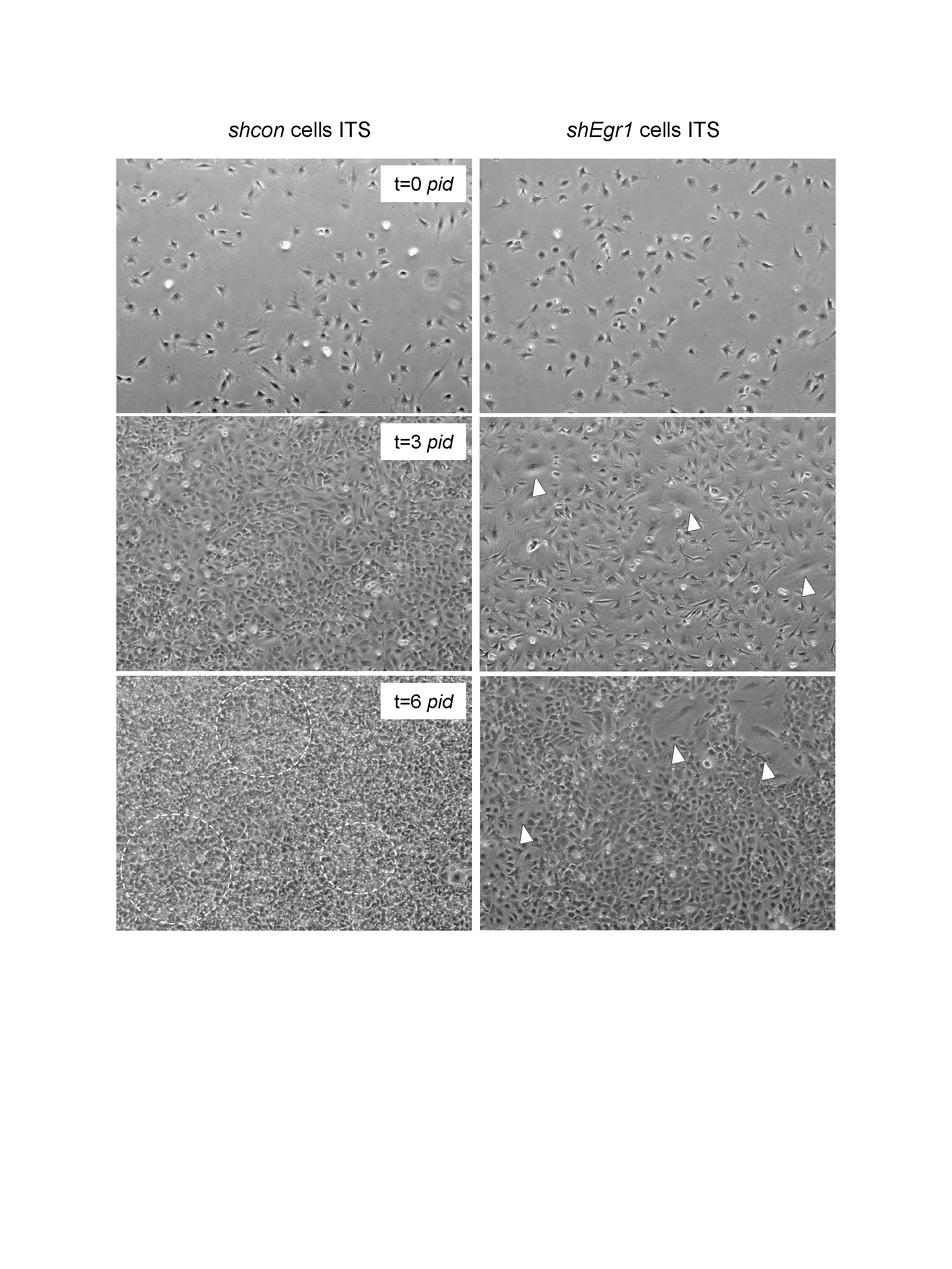

Supplement: Figure S7 — Loss of EGR1 affects differentiation induced hyper-proliferation. Morphological analysis of ATDC5 cultures stably expressing either shcon or shEgr1 vectors. Note that at 3 days pid differences in cell density are detectable and large flat cells appear (arrow heads); shEgr1 cultures do not reach super-confluence and do not form chondrogenic nodules (circles). (TIF) [file pone.0058083.s007.tif]

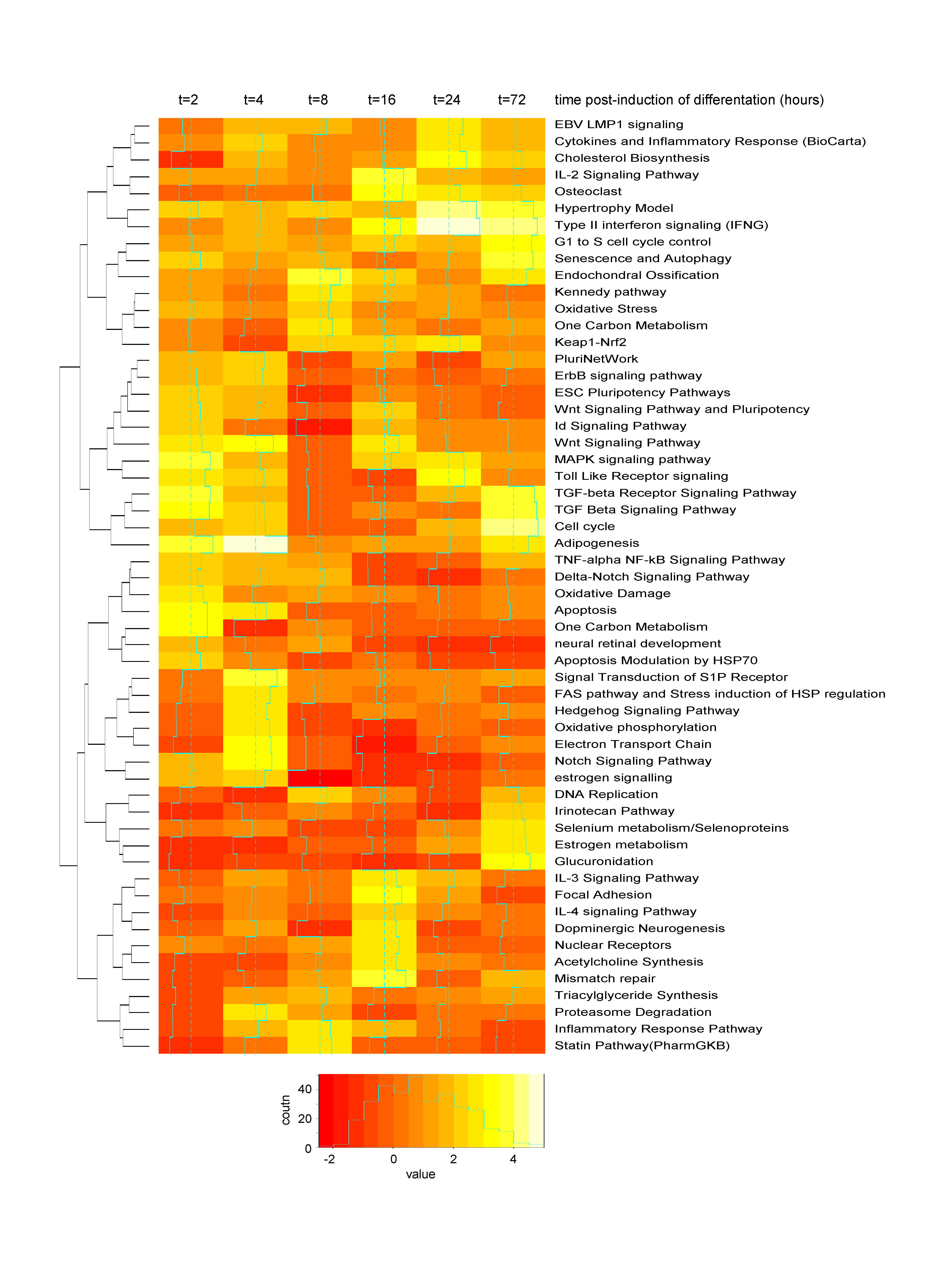

Supplement: Figure S8 — Deregulation of pathway in EGR1 deficient cultures. Pathway-heatmap of Z scores shEgr1 versus shcon. Pathways with a Z score of minimally 1.9 at any given time point are depicted; inset shows key to heatmap-colors (yellow: more genes/pathway, dark red: less genes/pathway deregulated in shEgr1 culture than expected on basis of random distribution) and histograms (percentage of genes deregulated in any given pathway). (TIF) [file pone.0058083.s008.tif]

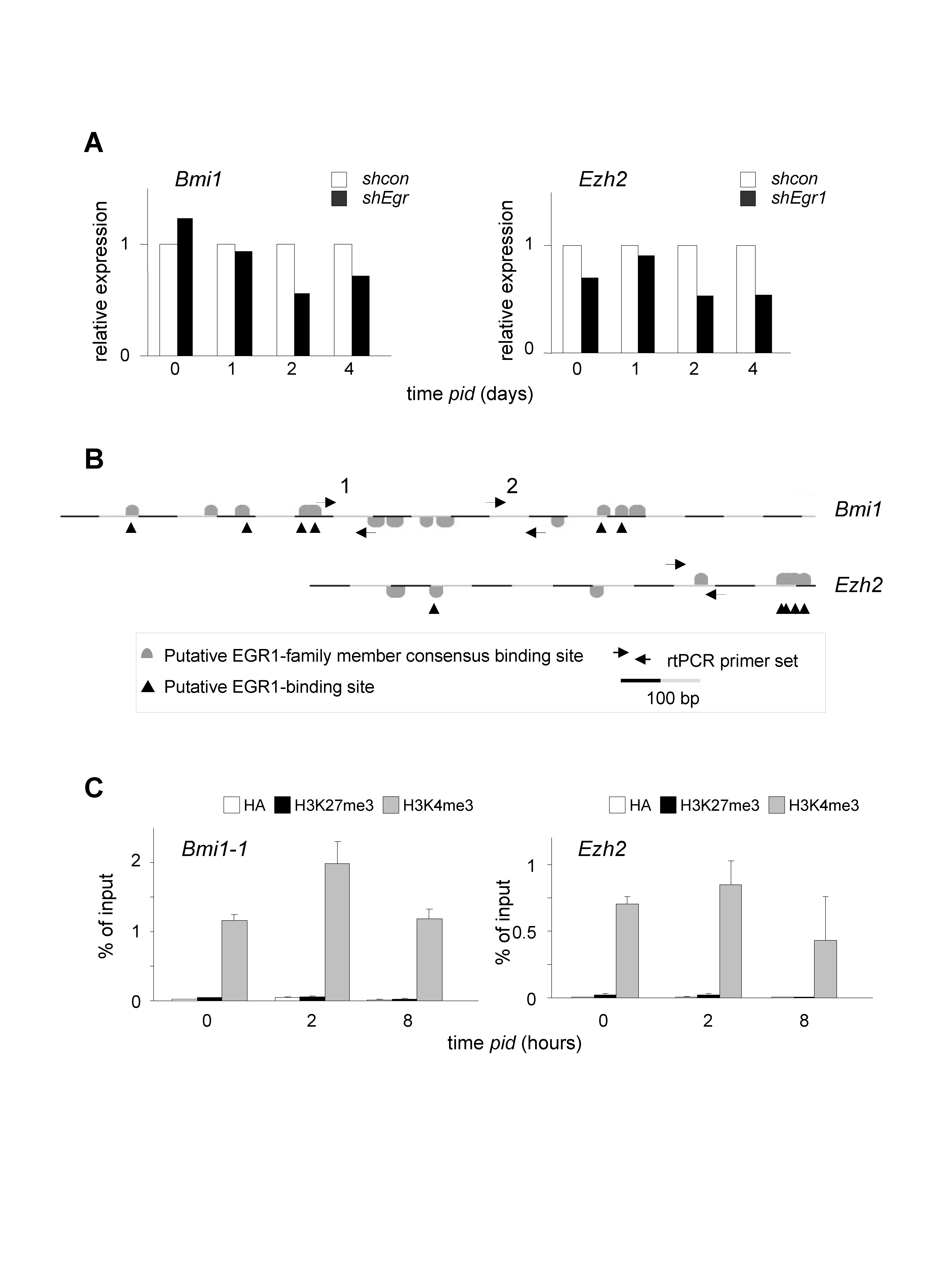

Supplement: Figure S9 — Reduced expression of PRC1 and PRC2-complex members BMI1 and EZH2 in shEgr1 -cultures. (A) Comparative mRNA expression of Bmi1 and Ezh2 in ATDC5 cells stably expressing shcon or shEgr1 as a function of differentiation. (B) promoter analysis of chondrogenic promoters for EGR1 binding sites (black triangles; GENOMATIX-based approach; see: Methods section); forward and reverse black arrows indicate primer locations for qPCR of immuno-precipitated chromatin. (C) H3K4me3 and H3K27me3-enrichment at Bmi1 and Ezh2 promoters at 0, 2 and 8 hours pid; control (con) ChIP experiments were carried out with a non-relevant haemagglutinin (HA) anti-serum. (TIF) [file pone.0058083.s009.tif]

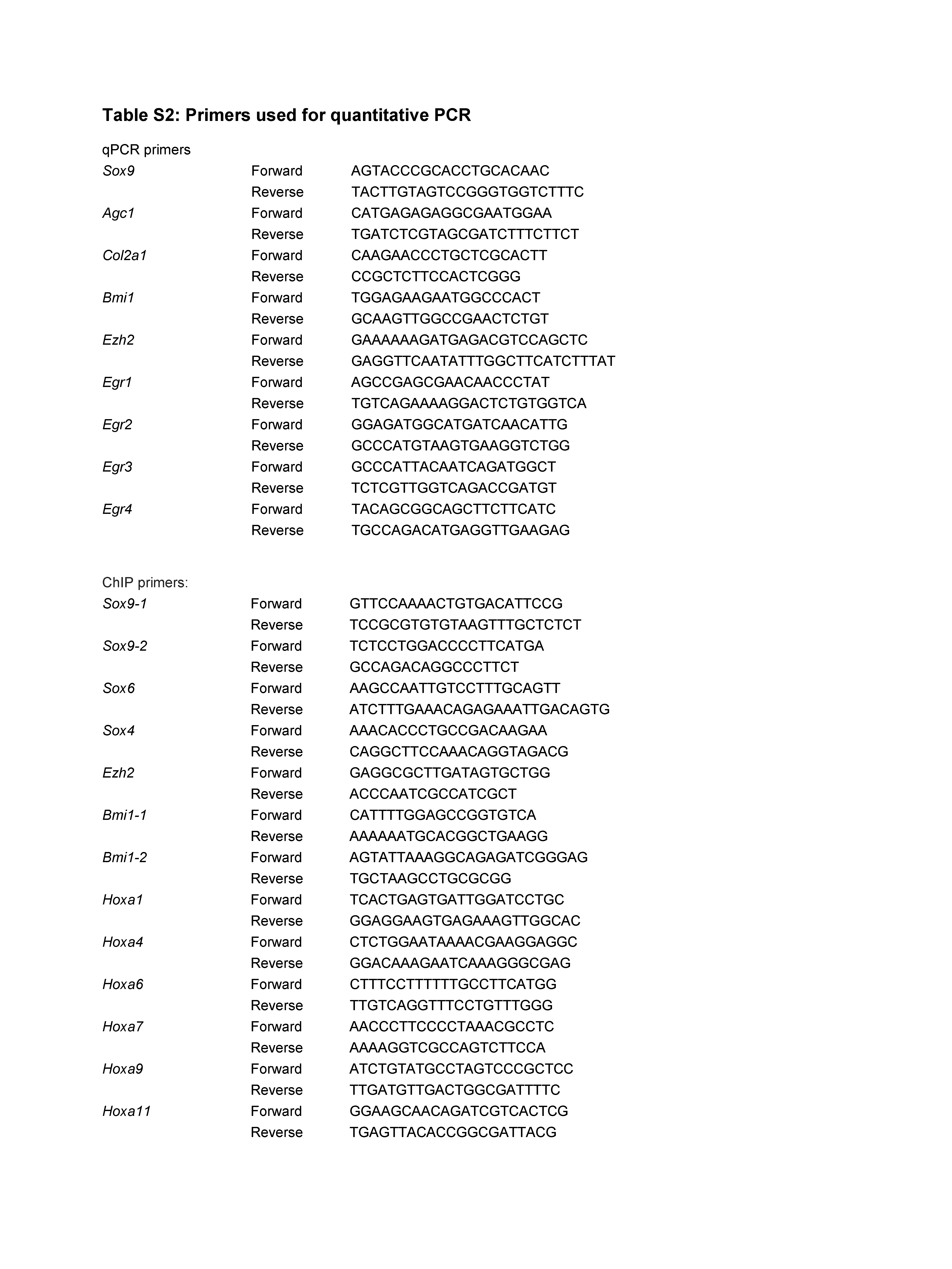

Supplement: Table S2 — Primers used for quantitative PCR. (TIF) [file pone.0058083.s011.tif]
